# Intra and interspecific audience effect on domestic dogs' behavioural displays and facial expressions

**DOI:** 10.1038/s41598-024-58757-6

**Published:** 2024-04-25

**Authors:** Giulia Pedretti, Chiara Canori, Elisa Costantini, Rupert Palme, Paola Valsecchi, Sarah Marshall-Pescini

**Affiliations:** 1 Department of Medicine and Surgery, University of Parma, Via Gramsci 14, 43126 Parma, Italy; 2 Department of Chemistry, Life Science and Environmental Sustainability, University of Parma, Viale Delle Scienze 17/A, 43124 Parma, Italy; 3 Domestication Lab, Konrad-Lorenz-Institute for Ethology, University of Veterinary Medicine, Veterinärplatz 1, 1210 Vienna, Austria; 4 Unit of Physiology, Pathophysiology and Experimental Endocrinology, Department of Biomedical Sciences, University of Veterinary Medicine, Veterinärplatz 1, 1210 Vienna, Austria

**Keywords:** Animal communication, Stress signals, Facial expressions, Visual signals, Domestic dog, Intraspecific communication, Interspecific communication, Audience effects, Displacement behaviours, Physiology, Animal behaviour

## Abstract

The aim of the current study was to investigate the influence of both intra- and interspecific audiences on dogs' facial expressions and behaviours. Forty-six dogs were exposed to three test conditions in which a food reward, initially available, was denied when in the presence of either a human (Human condition) or a dog audience (Dog condition), or in the absence of a visible audience (Non-social condition). Salivary cortisol was collected to evaluate the stress/arousal activation in the different conditions. Compared to the Non-social condition, the presence of a conspecific evoked more facial expressions, according to the DogFACS (Facial Action Coding System, an anatomically based tool to analyze facial expressions in domestic dogs), (EAD105—Ears downward), displacement behaviours (AD137—Nose licking, AD37—Lip wiping), tail wagging, whining, and panting (AD126). When facing a conspecific, dogs assumed a more avoidant attitude, keeping a distance and not looking at the stimuli, compared to when in the presence of the human partner. Dogs also exhibited more facial expressions (EAD102—Ears Adductor, EAD104—Ears Rotator), displacement behaviours (AD137—Nose licking, AD37—Lip wiping), panting (AD126) and whining when facing the conspecific than the human partner. Post-test cortisol was not influenced by any condition, and no association between pre-test cortisol and behavioural variables was found, thus strong differences in the levels of stress/arousal were unlikely to be responsible for differences in behavior between conditions. Considering the current results in the context of the available literature, we suggest that the higher displacement behaviors exhibited with the conspecifics were likely due to an increased level of uncertainty regarding the situations.

## Introduction

Communication, defined as a biological phenomenon in which the behaviour of an organism, the sender, directly or indirectly modifies the behaviour of another individual, the receiver, through the transmission of a signal^1^, has been intensively studied in many non-human species (in birds see^2,3^; in mammals see^4^). Signals have evolved through a ritualization process from precursor behaviours, which originally did not have a communicative function but were then gradually adaptively shaped in relation to the response of the audience (receivers of the signals)^5,6^. Audience effects on behaviour have been used to study the communicative and the intentional valence of behavioural displays in different species (e.g., Belding’s ground squirrels^7^; Gallus gallus^8^; Thomas langurs^9^; bonobos^10^), with dogs being one of them^11,12^.

Domestic dogs are highly social animals provided with a well-developed communication system which allows them to manage complex social interactions with both conspecifics and heterospecifics (such as humans)^13–15^ Communicative signals in dogs can be chemical, visual, auditory, or tactile^16,17^. Visual signals hold particular importance in short-distance interactions, and, in domestic dogs, they include body and tail postures^15,18^, facial expressions^11,12^ as well as displacement behaviours, i.e., patterns of behaviour exhibited outside of their functional context (e.g., lips licking, yawning, scratching, shaking, blinking^19,20^).

Facial expressions may have evolved as visual signals to maintain social cohesion, providing information about the sender’s emotional states (as suggested by the behavioural ecology view of facial displays^21^) and future actions^22^ to the receiver (studies in primates ChimpFACS^23^). Recent studies, analyzing dogs’ facial expressions with the DogFACS—The Dog Facial Action Coding System, have shown that their occurrence is influenced by the presence of an audience^11,12^. Pedretti et al.,^12^ showed that a number of facial expressions (i.e., EAD103—Ears Flattener, EAD101—Ears Forward), displacement behaviours (i.e., AD137—Nose Lick, AU145—Blink), as well as tail wagging during frustration evoking situations were dependent on the presence of a social partner (human experimenter) suggesting that dogs exhibit the above-mentioned behavioural displays as communicative visual signals towards their human social partner.

A few studies have also investigated which contexts may be more likely to elicit specific facial expressions and displacement behaviours. For example, Pedretti et al.^20^ found that yawning, blinking, lip wiping and nose licking were associated with a non-aggressive attitude of dogs towards an unfamiliar human approaching and were more likely to be exhibited when dogs were exposed to a video of a neutral panting dog than a threatening/barking dog^24^, leading authors to suggest that these behaviours may be related to the dog’s state of uncertainty regarding the situation. An aspect that has received relatively little attention so far, is whether dogs show similar behavioural signals towards a human and a conspecific in comparable situations.

The domestication process and co-habitation with humans has resulted in dogs showing a capacity to form attachment relationships with their caregivers^25–27^, indeed the relationship pet dogs exhibit with humans can be considered qualitatively comparable to the relationship they form with close conspecific partners^28^. In a context, in which food is out of reach, dogs appear to find it easier to cooperate with familiar human partners compared to familiar conspecific partners^29^, potentially because the competitive element over a food resource is absent with humans but a potential issue with conspecifics^30,31^ or because of the extensive reinforcement history of cooperation with humans that dogs undergo through their lives.

Not surprisingly then, dog–human communication has been observed in a variety of contexts: as following explicit human communicative cues such as pointing and verbal commands^32–34^, behaving in accordance with body orientation and attention^11,33^, as well as being sensitive to more subtle cues from their human handlers such as word meaning and intonation^35^, facial expressions^36,37^ and emotions through chemo-signals^15,38^. Furthermore, dogs have been shown to use attention-getting signals such as gazing and vocalizations to direct their owners’ attention to a desired object/resource^39–41^. However, to the best of our knowledge, only one study has directly compared the visual signals exhibited by dogs towards human and conspecific partners in an experimentally comparable situation.

Albuquerque et al.^36^ investigated the mouth-licking behaviour of dogs exposed to images of angry/aggressive human or dog faces/muzzles paired with either coherent or incoherent audio stimuli. Mouth-licking occurred more often when angry/aggressive visual stimuli were presented and more frequently when dogs were exposed to human than dog images leading the authors to conclude that mouth-licking could have been selected to facilitate dog–human communication. While dog–dog communication relies on all communicative channels (chemical, visual, auditory, and tactile), visual and auditory signals are the main source with which humans interpret dogs’ behavior, thus it is possible that dogs use different visual and auditory signals towards human and conspecific partners because of both selection during domestication and learning during a dog’s ontogenetic history^42^.

Given the paucity of studies on this topic, in the current study, we aimed to directly compare dogs’ use of visual signals when exposed to an unfamiliar human vs. an unfamiliar dog partner. Differently from Albuquerque et al.^36^, rather than using images of the two species, which may elicit a milder/less representative response, we adopted a ‘live’ experimental set-up. A frustration-evoking situation in which desired food was denied (thus likely producing a mildly negative state of arousal^43^) was presented to the dog subject in the presence of an unfamiliar human partner, an unfamiliar dog partner or in the absence of a visible audience (see Refs.^12,43^ for a similar setup). We monitored the potential influence of different stress/arousal levels on the likelihood of behavioural displays being exhibited in the three conditions by collecting salivary cortisol before and after each test condition.

As in previous studies, we used the ‘audience effect’ as a paradigm to investigate the communicative function of a behaviour^12,44^. In line with Pedretti et al.^12^, we predicted that displacement behaviours (i.e., AD137—Nose Lick, AU145—Blink), tail wagging, and a number of facial expressions (i.e., EAD 103—Ears Flattener; EAD101—Ears Forward) would be more likely to occur in the conditions where the audience (regardless of species) was present rather than in the absence of an audience. Furthermore, based on the only study comparing dogs’ communicative signals towards conspecifics and humans^36^, we tentatively predicted that dogs would show a higher likelihood of exhibiting mouth-licking (i.e., lip wiping or nose licking in the FAC system) when exposed to the human compared to the dog audience. However, this prediction was weighed against an opposite one, as we further posited that in the presence of food, dogs would perceive the conspecific near the food as a potentially competitive situation, thus increasing their level of uncertainty. In fact, in intraspecific contexts, dogs have been shown to have a low tolerance for food sharing^30,45^ and to adopt “conflict avoidance” strategies, whereby the lower ranking dog keeps its distance and avoids any confrontation with the higher-ranking individual [^31^; see^46^]. Thus, in the current study, we predicted that when faced with the conspecific audience close to the food, dogs would show more avoidant behaviours compared to when faced with the human audience, spending less time in proximity of the apparatus/food. Furthermore, we predicted that dogs would be more likely to exhibit head turning, blinking, yawning, lip wiping and nose licking with the conspecific than with the human, since these signals have previously been associated with an uncertain situation^20^.

## Results

All the dogs who participated in the assessment encounter were admitted to the test sessions.

Eleven DogFACS variables and six general behaviour variables were included in the statistical analyses since they satisfied the selection criterion (exhibited by more than 10% of subjects in at least one of the three test conditions—See Table 1). Furthermore, the time dogs spent staying in proximity of the apparatus and looking at the window were also analysed.

**Table 1:** Behavioural variables exhibited by > 10% of subjects in at least one condition and selected for the statistical analyses.

Ethogram	Category	Behaviour
DogFACS	Upper face Action Units (AUs)	Inner brow raiser (AU101)
		Blink (AU145)
	Mouth Action Descriptors (ADs)	Nose lick (AD137)
		Lip wipe (AD37)
	Ears Action Descriptors (EADs)	Ears forward (EAD101)
		Ears adductor (EAD102)
		Ears flattener (EAD103)
		Ears rotator (EAD104)
		Ears downward (EAD105)
	Gross behaviours	Panting (AD126)
General behaviours	Displacement behaviours	Head Turning
		Sniffing the environment
		Paw lifting
	Vocalizations	Whining
		Barking
	Other behaviours	Tail Wagging Looking at the window Proximity to the apparatus

### Effects of partner identity on behavioural responses

Human partner identity had no significant effect on subjects’ behavioural responses. Dog partner identity had no significant effect on most of the subjects’ behavioural responses except one: dogs spent more time close to the apparatus when the partner was Dog1 compared to Dog2 (Es = 0.625 ± 0.259, t-value = 2.413, *p* = 0.018).

### Effects of condition on stimuli window directed behaviours

Test condition had a significant effect on the time dogs spent close to (full-null model comparison χ^2^ = 71.067, *p* < 0.001) and looking at the stimuli window (full-null model comparison χ^2^ = 54.998, *p* < 0.001). Dogs spend less time close to the stimuli window in the Dog condition compared to both the Human (Es = 1.117 ± 0.185, z = 6.045, *p* = 0.001) and the Non-social condition (Es = 1.553 ± 0.185, z = 8.402, *p* = 0.001). Dogs spend less time looking at the stimulus window in the Dog condition compared to both the Human (Es = 1.2452 ± 0.164, z = 7.574, *p* < 0.001) and the Non-social condition (Es = 0.590 ± 0.164, z = 3.591, *p* < 0.001).

### Effects of condition on the likelihood of performing specific behaviours

**Figure 1:**
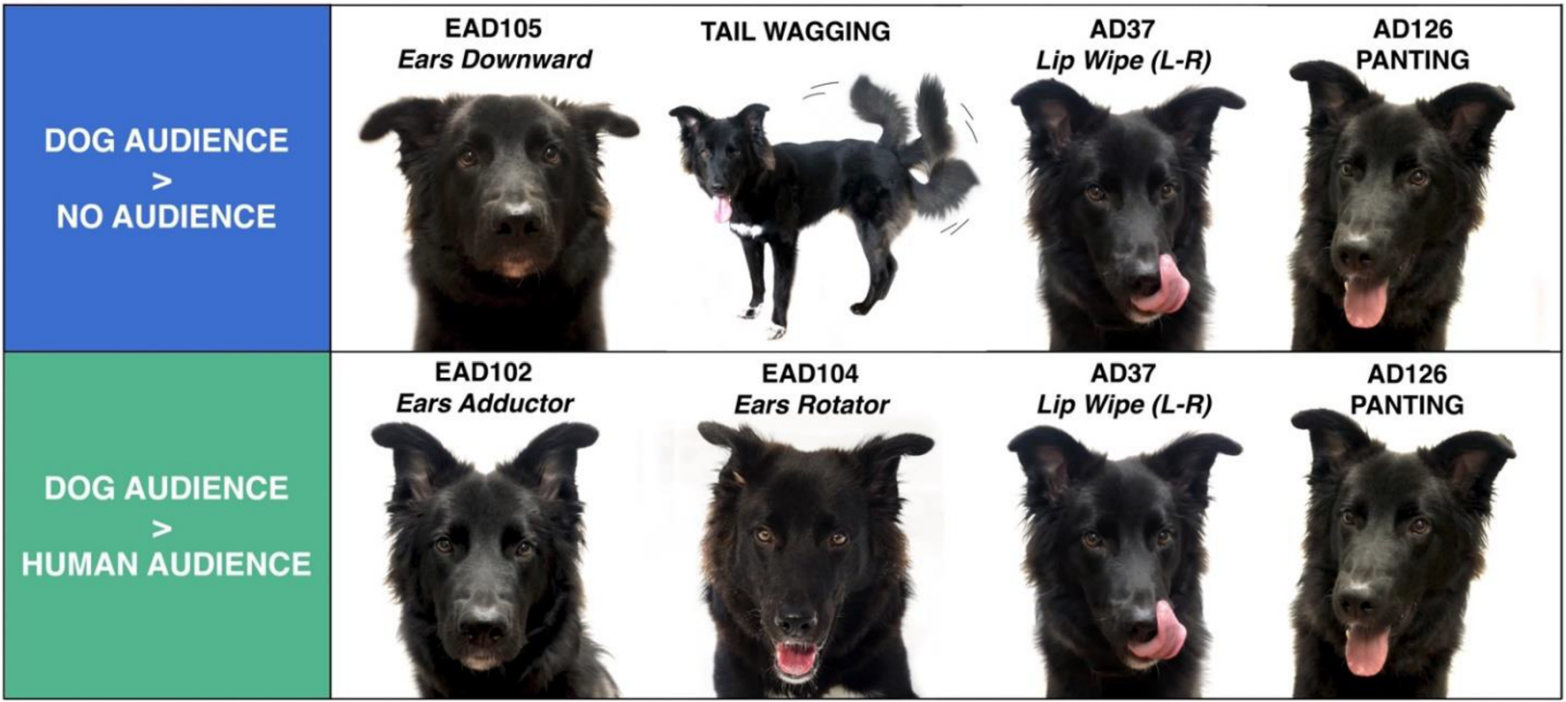
Behavioural variables that were more likely to occur 1st row: in the Dog partner compared to the Non-social condition and 2rd row: in the Dog partner compared to the Human partner condition.

Condition had a significant effect on nine behavioural variables. The likelihood of performing EAD101—Ears forward was higher in the Non-social condition compared to the Dog condition and higher in the Human compared to the Dog condition (full-null model comparison χ^2^ = 9.965, *p* = 0.007). The occurrence of EAD102—Ears adductor (full-null model comparison χ^2^ = 6.132, *p* = 0.047) was higher in the Dog condition compared to the Human condition. The likelihood of occurrence of EAD105—Ears downward (full-null model comparison χ^2^ = 6.742, *p* = 0.034) was higher in the Dog condition compared to the Non-social condition and the likelihood of performing EAD104—Ears rotator (full-null model comparison χ^2^ = 7.286, *p* = 0.026) was higher in the Dog condition compared to the Human condition. AD37—Lip Wipe, AD126—Panting and whining had a higher likelihood of occurrence in the Dog condition compared to both the Human and Non-social conditions (AD37: full-null model comparison χ^2^ = 12.948, *p* = 0.002; AD126: full-null model comparison χ^2^ = 35.145, *p* < 0.001; whining: full-null model comparison χ^2^ = 10.182, *p* = 0.006). Head turn (full-null model comparison χ^2^ = 7.502, *p* = 0.023) was more likely to occur in the Non-social compared to the Human condition. Tail wagging was more likely to occur in the Dog compared to the Non-social condition (full-null model comparison χ^2^ = 6.821, *p* = 0.033) (see Table 2 and Fig. 1 for estimated effects; see Supplementary materials Tables 4–21 for complete output of each statistical model including Confidence Intervals, and model Stability).

**Table 2:** Results of the post-hoc analysis on the likelihood of occurrence of the behavioural variables in the three test conditions.

Behavioural variable	Human audience effect (Human vs No audience)			Dog audience effect (No audience vs Dog)			Human audience compared to dog audience (Human vs Dog)		
	Estimate	Z	P	Estimate	Z	P	Estimate	Z	P
Ears forward (EAD101)	− 0.099 ± 0.223	− 0.447	0.896	0.636 ± 0.219	2.897	**0.011**	0.536 ± 0.218	2.463	**0.037**
Ears adductor (EAD102)	− 0.110 ± 0.210	− 0.526	0.858	− 0.399 ± 0.218	− 1.834	0.159	− 0.509 ± 0.212	− 2.350	**0.049**
Ears rotator (EAD104)	− 0.331 ± 0.211	− 1.570	0.259	− 0.228 ± 0.204	− 1.118	0.503	− 0.559 ± 0.209	− 2.671	**0.021**
Ears downward (EAD105)	0.482 ± 0.255	1.887	0.142	− 0.628 ± 0.254	− 2.474	**0.036**	− 0.146 ± 0.242	− 0.603	0.818
Lip Wipe (AD37)	0.055 ± 0.331	0.166	0.985	− 0.875 ± 0.289	− 3.204	**0.007**	− 0.820 ± 0.285	− 2.881	**0.011**
Panting (AD126)	0.602 ± 0.496	1.224	0.444	− 2.658 ± 0.544	− 4.811	** < 0.001**	− 1.978 ± 0.496	− 3.991	**< 0.001**
Head turning	− 0.051 ± 0.195	− 2.597	**0.025**	0.109 ± 0.191	0.574	0.834	− 0.396 ± 0.195	− 2.031	0.105
Tail wagging	0.648 ± 0.308	2.100	0.089	− 0.729 ± 0.307	− 2.374	**0.046**	− 0.082 ± 0.287	− 0.286	0.956
Whining	− 0.128 ± 0.357	− 0.357	0.932	− 0.822 ± 0.329	− 2.501	**0.033**	− 0.949 ± 0.336	− 2.828	**0.013**

Significant values are in bold.

### Effects of sex and morphological type

Results of the model considering only the dog partner condition revealed that the dog subject’s sex had a significant impact on the time spent near the apparatus (Es = 1.076 ± 0.258, t-value = 4.177, *p* < 0.001) and time spent looking at the stimuli (Es = 1.281 ± 0.361, z-value = 3.551, *p* = 0.000), with males spending more time close to the apparatus and looking at the stimuli compared to females. Furthermore, in male dogs the likelihood of performing AD37—lip wipe was higher compared to in females (Es = 1.129 ± 0.498, z-value = 2.604, *p* = 0.009).

Regarding the differences between the shepherd type dogs and the hunting type dogs, in hunting type dogs the likelihood of performing EAD105—Ears Forward (Es = − 1.659 ± 0.382, z-value = − 4.337, *p* < 0.001) and tail wagging (Es = − 2.087 ± 0.599, z-value = − 3.485, *p* < 0.001) was higher compared to shepherd type dogs.

### Association between behaviours and saliva cortisol

For 6 dogs, one or more of the 6 saliva samples did not contain enough saliva to measure cortisol concentration. Thus, 6 subjects were excluded from hormonal analyses and the results are based on 40 subjects, 17 females (12 spayed), 23 males (17 neutered). There was no effect of test condition on post-test cortisol concentration (full-null model comparison χ^2^ = 0.221, *p* = 0.895), however post-test cortisol levels were significantly related to pre-test cortisol concentrations (full-null model comparison χ^2^ = 106.160, *p* < 0.001), with dogs having higher pre-test cortisol concentrations also showing higher post-test cortisol concentration (see Table 22—Supplemental material). Pre-test cortisol concentration was not significantly associated with any behavioural variable (see Supplemental Material, Tables 22–38 for complete output of each statistical model including Confidence Intervals, and model stability).

## Discussion

The current study aimed to adopt a paradigm that would allow the investigation of potential differences in dog–human and dog–dog communication in a comparable setting using a ‘live’ audience. We exposed dogs to food denial in the absence of an audience, in the presence of an unfamiliar human experimenter and in the presence of an unfamiliar dog, both of which were looking at the food.

We found that the presence of a dog audience, compared to the absence of an audience positively affected the likelihood of a number of different facial expressions and behavioural displays i.e. ears downward (EAD105), lip wiping (AD37), panting (AD126), as well as tail wagging and whining. In addition, dogs were more likely to show a number of facial expressions i.e. ears adductor (EAD102), ears rotator (EAD104), lip wiping (AD37), and panting (AD126), as well as whining when faced with the conspecific, compared to the human partner.

Taken together, these results suggest that the facial expression displayed by the dogs do carry a communicative function, since they were indeed more likely to occur in the presence than in the absence of an audience. Furthermore, as post-test cortisol concentrations did not differ between experimental condition, it is unlikely that the differences in behavioural responses with the audience present was merely a result of a much stronger stress/arousal response in the audience condition. Results support previous finding in which dogs’ facial expressions, as well as tail wagging, were affected by the presence of an audience^12^. Interestingly, however, results from the current study do not entirely align with the previous study, in which the dogs’ facial expressions were analyzed as a function of the presence/absence of a human audience in the same food denial paradigm.

The previous study^12^ had found dogs exhibiting a higher frequency of ears forward (EAD101), ears flattener (EAD103), nose licking (AD137) and blinking (AU145), as well as tail wagging and whining in the presence vs. absence of a human audience. The current study did not find differences between the human audience and non-social control condition; this may be because in the current study the human experimenter looked at the food rather than looking directly at the dog. Dogs are sensitive to human attentional states^40^, thus it is possible that the gaze direction of the human was perceived as a lack of attention and hence affected the occurrence of the dogs’ facial expressions. Another possibility is that the person’s direct gaze in the previous study was interpreted as a mild threat, which hence elicited the dogs’ facial expressions and behavioral displays fulfilling an ‘appeasement’ or ‘submissive’ function.

Results from the current study also suggest that dogs’ facial expressions may be modulated by audience type i.e. whether it is a conspecific or a human. Both Ear Action Descriptors (EADs) (i.e. ears adductor (EAD102), ears rotator (EAD104)), as well as lip wiping (AD37) were more likely to occur in the presence of the conspecific audience than the human audience.

Previous studies have shown that the position of the ears is one of the main components of canids’ emotional expressions^15,43,47^. Bremhorst and colleagues^47^ showed that ears adductor (EAD102) was more likely to occur during a positive experience while ears downward (EAD105) was more likely to occur during a more negative experience. Lip wiping (AD37) and nose licking (AD137) was previously found to be an indicator of positive emotion in one study^48^, but other results showed that nose licking (AD137) was more common in an emotionally negative condition compared to a positive one^43^. In a third study, both displacement behaviours were associated with a non-aggressive attitude of dogs towards an unfamiliar human approaching^20^ and were exhibited more frequently when dogs were exposed to a video of a neutral dog than an threatening/barking one^24^. Thus, considering the varied results from the literature, it is difficult to draw conclusions in relation to the specific function of the facial expression observed. However, in the current study, several additional behavioural differences between conditions may give some indications.

Indeed, dogs in the Dog test condition spent less time in proximity to the opening and were more likely to turn their head away, pant and whine than in the Human audience condition. The latter (panting and whining) are behaviours which have been associated with negative emotional states^49,50^, whereas head turning (or gaze avoidance) has been considered as a submissive behaviour^51^. Thus, taken together, results would suggest that dog subjects balanced a higher interest/alertness during the conspecific audience condition (e.g. ears adducted) with a potentially higher level of uncertainty, exhibited with an increase in avoidance (i.e. distance maintenance and head turning) and a higher likelihood of putative appeasement signals such as lip wiping. These results are in line with studies showing that dogs will largely enact conflict avoidance strategies in intraspecific contexts involving food^30,31^ even when the ‘conspecific’ is merely an image of another dog projected in such a way to appear to be looking at the food source^52^.

Albuquerque et al.^36^ found that dogs licked their mouths more frequently when pictures of angry human faces were shown to them, rather than towards pictures of angry dog faces. As neither the human nor dog stimuli in our study were openly threatening, or showed angry expressions during the test, the dogs tested may not have perceived the human partner as a sufficiently confrontational stimulus, whereas a still conspecific may have been sufficiently threatening to support a higher occurrence of lip wiping behaviour (AD37) in the 'Dog' audience test condition.

Indeed, a promising future avenue of research would be to adopt the same paradigm used in the current study varying the emotional expression of the audience which may help to disentangle the putative appeasement function of specific facial expressions.

A number of limitations of the current study need to be considered. Firstly, both the human and dog audiences were all female, and we have some indication that this may indeed have influenced the dogs’ responses. In fact, when considering the dog partner condition, our results showed that male subjects were more prone to stay in closer proximity to the apparatus, look at the conspecific more and perform a higher frequency of lip wiping. These results would suggest that male dogs were somewhat less intimidated by the presence of the opposite sex conspecific close to a food source, which is in line with findings from other experimental paradigms^53^. Nevertheless, this aspect will require further investigation in future studies.

A second aspect is that although we pre-selected subjects excluding all dogs that showed any signs of discomfort in the presence of the audience dog prior to the test and we included cortisol measures to assess/control for potentially large variations in stress measures between conditions, we cannot completely exclude the possibility that the behavioural differences exhibited reflect a generalized increase in the dogs’ arousal/stress levels. Of course, facial expressions and behavioural signals, may be both a result of the internal state of the animal and fulfill a communicative function, however, at present we cannot fully disentangle these two factors. In fact, dogs’ responses to the vision of conspecifics near food, even if not clearly aggressive, could have been affected by their previous experience interacting with other dogs, factor not controlled in this experiment.

In conclusion, to the best of our knowledge, this is the first study investigating the effects of a conspecific vs. human audience on dogs’ behavioural displays by using a ‘live’ audience in a directly comparable setting. The experimental design and paradigm adopted revealed that the conspecific audience elicited an increase in the display of specific behaviours (i.e. EAD102—ears adductor, EAD104—ears rotator, AD37—lip wiping), consistent with the hypothesis that these behaviours are displayed in situations of increased uncertainty.

## Materials and methods

### Ethical statement

The experimental procedure was approved by the Animal Welfare committee of the University of Parma in accordance with Animal Welfare organization committee of the University of Parma (OPBA) (Protocol number PROT. N. 6/CESA /2022) and ARRIVE guidelines and regulations. Owners signed informed experimental procedure consent and privacy consent for publishing images and findings.

### Subjects

We tested 46 subjects, 23 males (3 neutered) and 23 females (17 spayed), ranging from 1 to 11.5 years (mean = 4.1 ± 2.9 years). Since facial morphology may affect facial expressions^12^ subjects were divided into two categories based on their facial morphology: shepherd-type dogs with erect ears and triangular snouts (n = 27) and hunting-type dogs with floppy ears and a square face (n = 19). None of these dogs participated in our previous study^12^. Based on the owners’ reports, none of the subjects presented sensory deficits or other diseases. Owners were asked to avoid training and feeding the dog for 2 h and drinking 15 min before the test.

### Experimental design and set-up

The tests were conducted at the Dog’s Ethology Lab of the University of Parma, between March 2022 and July 2022. We adopted a within-subject design, so each dog came to the laboratory four times on different days, at the same time of day to control for cortisol circadian rhythm fluctuations^54^. The test was conducted in an indoor room (4 × 7 m) divided by a wooden apparatus with a 50 × 100 cm open window in the middle at 30 cm height from the floor (see^12^ for a detailed description of the experimental set-up). The window was covered by two movable panels: a plexiglass one and an opaque one. A table was placed directly behind the window, on which a stick with a plate attached could be moved by an experimenter positioned behind the apparatus (out of sight of the subject). A dog crate was placed behind the apparatus (out of sight of the subject), in which the dog partner could rest during the intraspecific audience test sessions. Four cameras were connected to the computer allowing us to record the subject and the stimulus.

Two female human partners and two female dog partners were used as the ‘audience’ for the tests. Half of the dogs were tested with Human 1 and half of the dogs with Human 2. The dog partners were Dog 1, a 7-year-old Weimaraner (spayed), and Dog 2, a 2-year-old Rhodesian Ridgeback (not spayed). Prior to the start of the tests, the dog partners were trained to sit still and look at the food reward placed on the table in front of them until the experimenter closed the opaque panel and then delivered the food reward (thus dogs were never allowed to independently take the food from the plate). Both the human partners and the dog partners were all unfamiliar to the dogs tested. Twenty-four dogs were tested with Dog 1 and twenty-two with Dog 2.

### Experimental procedure

One assessment session and three test sessions were carried out on different days, each session lasting approximately 30 min.

During the assessment session each potential dog subject was evaluated and, if suitable (see below), was admitted to the three following test sessions. During the assessment session, the potential dog subject met the dog partner in a large meadow near the test room. Both dogs were kept on a leash during the whole encounter, and they were always kept at 10-m distance from one another. A dog subject was considered suitable if it did not show aggressive or fearful behaviours towards the dog partner. If the dog was considered suitable, the experimenters and the dog partner preceded the subject and the owner into the test room and waited for them behind the apparatus (where the dog partner was placed in a dog crate). Upon the owner’s and the dog’s arrival, the owner was asked to unleash the dog, allowing it to roam freely, explore the environment, and familiarize itself with the experimental setup. The owner was asked to sit, wear a face mask and sunglasses, and to ignore the dog for the duration of the session. The dog subject was then presented with 10 trials in which it was allowed to retrieve a piece of food from the table behind the window (task-acquisition phase). The procedure began with both panels covering the window, then the opaque panel was opened and, after 3 s, the Plexiglas panel was opened, thereby allowing the subject to take the food. The experimenters and the dog partner were not visible during this procedure. After the first 10 trials, 2 additional trials were performed. In these trials, the food reward was denied (moved away from the window), the transparent panel remained closed, and, behind the window, the dog partner sat staring at the food, in full view of the dog subject. If the dog subject had high food motivation (i.e., took all the food items in the 10 trials staying in front of the window when the Plexiglas was closed) and did not show excessive aggressive behaviour when the dog partner was visible (i.e., repeated barking and growling), then the dog subject was allowed to participate in the three test sessions.

In the three test sessions, which took place on three different days and were presented in a counterbalanced order across subjects, the experimenter -or the experimenter with the dog partner—met the dog subject and owner outdoors and then entered the test room together. The experimenter then collected the first saliva sample, stored it in the freezer (at − 20 °C). The dog subjects always started the session with 10 trials in which they could obtain the food (see task acquisition phase described above).

**Figure 2:**
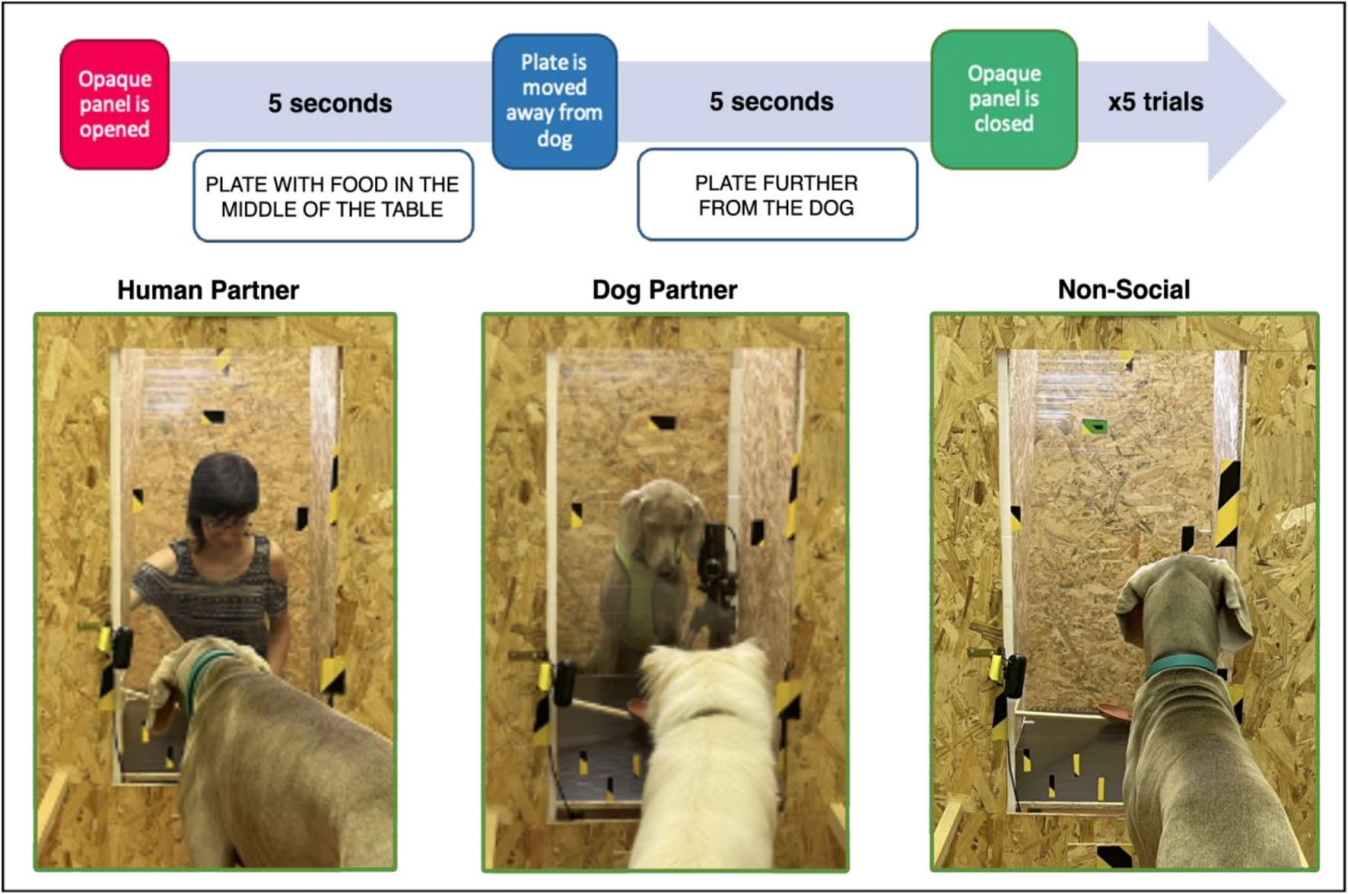
Images from the three frustration test conditions and timeline for the experimental test trials.

The test session consisted of 3 conditions and a total of 5 consecutive trials per condition, each trial lasting 10 seconds (see Figure 2 for the timeline of the trials).

- **Human partner test condition (Human):** Five food denial trials with a visible experimenter looking at the food. The food reward was placed on the plate out of sight of the dog subject, after opening the opaque window, the tested dog could see the plate with the food reward and the experimenter looking at the food for 5 s. The reward was then moved away from the window to a position closer to the experimenter, remaining unreachable for a further 5 s. Finally, the opaque panel was closed. This procedure was repeated 5 times.
- **Dog partner test condition (Dog):** Five food denial trials with a visible conspecific looking at the food. The food reward was placed on the plate out of sight of the dog subject, after opening the opaque panel, the tested dog could see the plate with the food reward and the dog partner staring at the food for 5 s. The reward was then moved away from the window and closer to the dog partner, remaining unreachable for a further 5 s. Finally, the opaque panel was closed. This procedure was repeated 5 times.
- **Non-social test condition (Non-social):** Five trials in which food was denied, with no audience visibly present. The food reward was placed on the plate out of sight of the dog subject, after opening the opaque panel, the tested dog could see the plate with the food reward for 5 s. The reward was then moved further away from the dog, remaining visible but unreachable for a further 5 s. Finally, the opaque panel was closed. This procedure was repeated 5 times.

At the end of each test, the owner and the subject waited in the experimental room for 15 min (cortisol reactivity time—Chmelíková et al.^54^) until the second (post-test) saliva sample was collected.

### Hormonal measures and laboratory analysis

As in Pedretti et al.^12^, saliva samples were collected with Salivettes (Sarstedt, Ges.mbH, Wr. Neudorf, Austria) to measure cortisol concentration (i.e., a validated physiological indicator of stress in dogs^55^). Saliva was collected by inserting the swab inside the dogs’ mouths for a few seconds, until it was saturated. Pre- and post-test saliva samples were collected for each subject in every test condition. All samples were named, numbered, dated, categorized as “pre” or “post” test, put in a small bag labelled with the subject’s name (along with all the other samples collected from the same dog) and stored at − 20 °C. They were then thawed at room temperature for 30 min and centrifuged for 15 min at 20 °C at 1500 rpm. A standard cortisol enzyme immunoassay^56^ was used to analyse cortisol levels.

### Behavioural coding

Tests were recorded by three video cameras synchronized with OBS software (https://obsproject.com) and Solomon Coder Beta 15.01.2013 (Andrá Péter, http://solomoncoder.com) was used for behavioural coding. Dogs’ facial expressions were coded based on DogFACS (Facial Action Coding System)^43,48,57,58^ (see Table 2 and Table 3—Supplementary material—for a detailed ethogram). A second ethogram including position, proximity to the window, gaze orientation, vocalization, and displacement behaviours (self-directed and environment-directed) was used to code general behaviours (See Table 4—Supplementary material for detailed ethogram). Behavioural variables were coded as present or absent as well as in terms of their duration or frequency in each 5 s trial. Videos were coded by two certified DogFACS experimenters (EC and CC). Both coders analysed 72 videos (20% of the videos) to assess inter-rater reliability. Intra-class correlations (ICCs) were performed in R 4.1.0 (function: “ICC”; package: psych). Ears Action Descriptors, Displacement behaviours and Vocalization had a moderate strength of inter-rater agreement (EADs and Events: 0.70 < ICC < 0.75), all the other variables showed an excellent reliability (0.85 < ICC < 0.95).

### Statistical analyses

For the statistical analyses, we selected DogFACS units and behaviours observed in more than 10% of the subjects in at least one of the three test conditions. Generalized linear mixed models (GLMM) were used to analyse the behavioural variables. Based on previous literature^12,43^ most behavioural variables were analysed in terms of the likelihood of their occurrence (presence or absence) in each trial (binomial model). For two variables i.e. ‘staying in proximity’ and ‘looking at the window’ the duration of the variable in each trial was used as the response variable.

A preliminary analysis was carried out to assess whether the dog and human partner identity (Dog1 and Dog2, CC and EC) affected the subjects’ response. Thus, we ran two separate models (one for the Human and one for the Dog condition) including the identity of the dog or human partner as the predictor variable, trial number and sex as fixed factors and age of the subject as covariate.

To test the effect of the presence and type of audience (dog or human partner) on the subjects’ behavioural responses, models were performed using the function “glmmTMB” of the package “glmmTMB”. Test condition (human, dog, non-social) was entered as the predictor variable, trial number, dog type (hunting or shepherd type) and sex were entered as fixed factors, age was entered as a covariate while subject ID was included as a random factor to control for repeated sampling. To keep the type I error rate at 5%, as an overall test of the effect of the predictor variable on the response variable^59^, we compared the full model with a null model lacking the predictor variable of interest (i.e. “condition” or “trial number”) using a likelihood ratio test^60^. Post Hoc pairwise comparisons between test conditions (Non-social and Human, Non-social and Dog, Dog and Human) were performed with Tukey contrast using the function “glht” of the package “multcomp”. We assessed model stability on the level of the estimated coefficients and standard deviations by excluding the levels of the random effects one at a time. This revealed the models to be of good stability (see Supplemental Materials for detailed results). Parametric bootstrapping was performed to obtain confidence intervals (function “boot.glmmTMB”).

Regarding the hormonal measures, we first assessed whether the different test conditions influenced the post-test saliva cortisol concentration. We performed a GLMM with Gaussian error structure using the function “glmmTMB” of the package “glmmTMB”. Post-test cortisol concentration was used as response variable while test condition was entered as the predictor. Pre-test cortisol concentration and sex of the subject were included as fixed effects, age of the subjects as a covariate while subject ID as random factor. Since post-test saliva cortisol concentration was significantly influenced only by pre-test saliva concentration and not by the different test conditions, we then investigated whether pre-test saliva cortisol concentration was associated with behavioural variables exhibited during the tests. Thus, we ran GLMMs with either the durations or the frequencies of the behavioural variables as the response variable, test condition and pre-test cortisol saliva as the predictor variables, sex, and dog type were included as fixed effects, and age as a covariate. Subject ID (repeated observations of the same individual) and plate ID (cortisol samples were analysed in 8 plates containing 6 subjects each) were included as random factors. We assessed model stability and parametric bootstrapping was performed to obtain confidence intervals (function “boot.glmmTMB”).

All statistical analyses were performed in R (version 3.6.1; R Core Team 2019). Results were considered statistically significant when p ≤ 0.05.

### Supplementary Information

Supplementary Tables.

## Data Availability

The raw data and analysis of this study are available from the corresponding author on request.
