# Supplementary material for: Intra and interspecific audience effect on domestic dogs' behavioural displays and facial expressions
Source: Sci Rep. 2024 Apr 25;14:9546. doi: 10.1038/s41598-024-58757-6 (PMC11045831; doi:10.1038/s41598-024-58757-6)
Supplement: Supplementary file 1 — Supplementary Tables. [file 41598_2024_58757_MOESM1_ESM.docx]

**Supplemental Material**

**Intra and interspecific audience effect on domestic dogs' behavioural displays and facial expressions**

Table S1 – Subjects who participated in the study.

| **subject** | **dog_type** | **Breed** | **age** | **sex** | **neutered** | **experimenter** | **dog_stimuli** |
| --- | --- | --- | --- | --- | --- | --- | --- |
| achille | hunting_type | golden retriever | 5 | m | no | EC | Dog2 |
| aki | hunting_type | mix breed | 5.5 | m | yes | EC | Dog2 |
| aramis | sheperd_type | belgian malinois | 4.5 | m | no | CC | Dog1 |
| ares | sheperd_type | german shepherd | 4.5 | m | no | CC | Dog2 |
| bella | sheperd_type | german shepherd | 4.5 | f | no | EC | Dog2 |
| buddy | hunting_type | mix labrador retriever | 10 | m | yes | CC | Dog1 |
| cake | sheperd_type | Australian cattle dog | 4.5 | m | no | CC | Dog1 |
| camilla | sheperd_type | Australian cattle dog | 4.5 | f | no | EC | Dog1 |
| caspian | hunting_type | Dobermann | 1 | m | no | EC | Dog2 |
| castiel | sheperd_type | Siberian Husky | 4.5 | m | no | CC | Dog1 |
| chacha | hunting_type | golden retriever | 8 | f | yes | EC | Dog2 |
| cheube | hunting_type | Lagotto romagnolo | 2 | m | no | CC | Dog1 |
| dalia | sheperd_type | german shepherd | 4.5 | f | yes | CC | Dog1 |
| dike | sheperd_type | mix Belgian Malinois | 4.5 | f | no | CC | Dog1 |
| dylan | hunting_type | mix Drahthaar | 3 | m | no | EC | Dog2 |
| esh | hunting_type | mix pitbull | 1 | f | yes | CC | Dog1 |
| eva | hunting_type | italian hound | 9 | f | yes | EC | Dog2 |
| fiamma | hunting_type | vizsla | 2 | f | no | EC | Dog2 |
| giotto | hunting_type | Lagotto romagnolo | 8 | m | no | EC | Dog1 |
| grace | hunting_type | labrador retriever | 5 | f | no | EC | Dog2 |
| gus | sheperd_type | belgian malinois | 4.5 | m | no | EC | Dog2 |
| kira | sheperd_type | german shepherd | 4.5 | f | yes | CC | Dog1 |
| kobe | hunting_type | flat coated retriever | 1 | m | no | EC | Dog2 |
| kronos | sheperd_type | german shepherd | 4.5 | m | no | CC | Dog1 |
| kylo | sheperd_type | german shepherd | 4.5 | f | yes | EC | Dog1 |
| leone | sheperd_type | Chow Chow | 4.5 | m | yes | CC | Dog1 |
| lilly | hunting_type | labrador retriever | 5 | f | yes | EC | Dog2 |
| link | sheperd_type | Australian shepherd | 4.5 | f | yes | CC | Dog1 |
| ludwig | sheperd_type | border collie | 4.5 | m | no | EC | Dog1 |
| luna | hunting_type | mix golden retriever | 10 | f | yes | CC | Dog1 |
| marek | sheperd_type | german shepherd | 4.5 | m | no | EC | Dog2 |
| maya | sheperd_type | Siberian Husky | 4.5 | f | yes | CC | Dog1 |
| mica | sheperd_type | belgian malinois | 4.5 | f | no | EC | Dog2 |
| milo | sheperd_type | german shepherd | 4.5 | m | no | EC | Dog2 |
| misaki | sheperd_type | akita inu | 4.5 | m | no | EC | Dog1 |
| mutt | sheperd_type | belgian malinois | 4.5 | m | no | CC | Dog1 |
| nerone | sheperd_type | Australian shepherd | 4.5 | m | no | EC | Dog2 |
| oliver | hunting_type | Lagotto romagnolo | 8 | m | no | EC | Dog2 |
| olympia | sheperd_type | german shepherd | 4.5 | f | yes | EC | Dog2 |
| rita | sheperd_type | german shepherd | 4.5 | f | yes | CC | Dog1 |
| selli | sheperd_type | german shepherd | 4.5 | f | yes | EC | Dog2 |
| stella | hunting_type | weimaraner | 2 | f | yes | EC | Dog1 |
| sunny | hunting_type | vizsla | 1 | f | no | CC | Dog1 |
| sunny_2 | sheperd_type | swiss shepherd | 4.5 | f | no | CC | Dog1 |
| timmy | sheperd_type | border collie | 4.5 | m | no | EC | Dog2 |
| zelda | hunting_type | mix breed | 1.5 | f | yes | EC | Dog2 |

**Table S2** – DogFACS ethogram.

| **General Category** | **Category (mutually exclusive behaviours)** | **Code** | **Definition** | **Description** | **Type of measure** |
| --- | --- | --- | --- | --- | --- |
| ACTION UNITS (Facial movements) | ACTION UNITS (AU) - Upper Face | AU101  (R-L) | Inner Brow Raiser | When the inner brow raises it can be distinguished a dorsal movement of the protuberance on the inner eye. The dog raises the soft protuberance present above the inner corner of the eye. Eyes become rounder and wider, and a vertical wrinkle may appear just between them. The left and the right region of the brow can be moved separately (**AU101-R** or **AU101-L**) or together (**AU101**). | Duration |
|  |  | AU143 | Eye Closure | Upper and lower eyelids move towards together until they completely touch each other when the eyes close completely. Eyes remain closed for **at least half a second**. | Duration |
|  |  | AU145  (R-L) | Blink | Upper and lower eyelids move towards together until they completely touch each other when the eyes close completely. In the AU145 **eyes open again within half a second**. The left and right eye can be closed indipendently (lateralized movement) (AU145-R, AU145-L) or together (AU145). | Event |
|  | ACTION UNITS (AU) - Lips Lower Face I | AU109+110 | Nose wrinkle and Upper lip raiser | In dogs the nose wrinkle (**AU109**) is only observed with AU110 (upper lip raiser) since they have only three muscles (levator nasolabialis, caninus, levator labii maxillaris) that work together for raising the upper lip and pulling the nose dorsally and/or caudally. These movements usually induce wrinkles formation on the muzzle and often come together with a snarl, causing the display of upper teeth.  The upper lip raiser (**AU110)** can also be seen without the nose wrinkle (AU109), hence to the raise of the levator nasolabialis muscle alone.  It can be observed with AU26 or AU27. | Duration |
|  |  | AU110 | Upper lip raiser |  | Duration |
|  |  | AU12 | Lip corner pull | Due to the contraction of the zygomaticus muscle the lips' corners are pulled towards the ears, curving slightly.  The mouth is opened, more teeth are visible, and some wrinkles may appear around the lips' corners as they're stretched. | Duration |
|  |  | AU118 | Lip pucker | The dog pushes the corners of its mouth forward (rostrally), the muzzle stays tense and some teeths may be visible if the mouth slightly opened. | Duration |
|  | ACTION UNITS (AU) -Lips Lower Face II | AU116 | Lower lip depressor | Contrary to the neutral state with mouth open, in whom only the tips of the canines are visible, this movement makes other lower teeth, sometimes even the gums, visible as the lower lip's withdrawn ventrally. | Duration |
|  | ACTION UNITS (AU) - Lower Face III | AU26 | Jaw Drop | The lower jaw is dropped without any sign of tension (mouth sglightly open).  **AU26** can been coded even without a clear sign of lips separation, but teeth separation must be seen or at least perceived. | Duration |
|  |  | AU27 | Yawning – Mouth Stretch | The mouth is stretched opened and the lower jaw tensed (mutually exclusive of the AU26). Teeth are exposed, the tongue and the oral cavity are shown and the lips are pulled back. | Event |

**Table S3** – General behaviours ethogram.

| **General Category** | **Behavior** | **Description** | **Type of measure** |
| --- | --- | --- | --- |
| Position | Stand | The dog stand with all four paws on the ground and does not move. | Duration |
|  | Sit | Stationary position, with only the backside in contact with the ground. | Duration |
|  | Lay | Dog's belly touches the ground as it lays completely down on the floor. | Duration |
| Locomotion | Back | The dog walks backwards. | Duration |
|  | Freeze | Reactive immobility. The body is completely rigid, while the dog keeps staring the stimuli (or the apparatus). | Duration |
|  | Still | Absence of movement, differently from freezing the dog's body is not rigid, but kept in a fixed posture. | Duration |
|  | Trot | The dog is trotting round the room. | Duration |
|  | Walking | The dog is walking. | Duration |
| Body Orientation | Door | The dog has its body directed towards the exit door. | Duration |
|  | Owner | The dog has its body directed towards the owner. | Duration |
|  | Set-up | The dog has its body directed towards the apparatus. | Duration |
|  | Stimuli | The dog has its body directed towards the stimuli. | Duration |
| Head | Face Down | The dog keeps its head downwards towards the floor. | Duration |
|  | Door | The head of the dog is directed towards the exit door. | Duration |
|  | Owner | The head of the dog is directed towards the owner. | Duration |
|  | Set-up | The head of the dog is directed towards the apparatus (excluded the stimuli window) | Duration |
|  | Stimulus (looking at the window) | The head of the dog is directed towards the stimuli window. | Duration |
|  | Turn left | The dog is looking at the stimuli's window (food or experimenter) and then turns the head to its left. Looking away from the stimuli (food/experimenter) | Duration |
|  | Turn right | The dog is looking at the stimuli/the apparatus and then turns the head to its right. Looking away from the stimuli (food/experimenter). | Duration |
| Wagging | wag | The dog moves its tail back and forth at any speed or direction | Duration |
| Proximity to the apparatus | Close (Proximity to the apparatus) | The dog has at least one paw in the area delimited by the two wooden bars or is in contact with them and oriented towards the stimulus | Duration |
|  | near | The dog is outside the area delimited by the two wooden bars, not in contact with them, but neither in contact with the owner, the door or the walls. | Duration |
|  | far | When the dog is in the area delimited by the owner chair, the black table and the door + near the wall outside the field of view of the stimulus. | Duration |
| Events | Autogrooming | The dog starts licking and wiping its own body in order to clean it. | Duration |
|  | Drinking | Whenever the dog starts drinking water from the bowl. | Duration |
|  | Head_turn | The dog is looking at the stimuli window or at the experimenter (in FH) and averts its gaze moving the head towards the right or the left. | Event |
|  | Paw lifting | The dog lifts one of the front paws, even slightly. | Duration |
|  | Scratching | The dog scratches repeatedly its neck or face with the hind legs. | Duration |
|  | Shaking | Dog's whole body and head starts moving rapidly, from side to side. | Event |
|  | Sniffing Environment | Dog's muzzle gets closer to the floor, the exit room, or other items inside the experimental room, while its nose snuffles it and nostrils moves quickly. | Duration |
|  | Stretching | The whole body is stretched in various ways: forelegs may be leant down while the dog's back is arched; hind legs may be straightened while the head is held up high. | Event |
| Vocalizations | Whine | The dog starts vocalizing, making persistently whines. | duration |
|  | Growl | Snarling sound directed at something or as a reaction to something in the living environment. Mouth may be open; nose can be wrinckled. | duration |
|  | Bark | Loud and short typical vocalization, which may be repetitive. | duration |

**Tables reporting the complete output of each statistical model including Confidence Intervals, and model Stability.**

In all the models the the reference level for the Fixed factor **Condition** (FD- Frustration dog/FH – Frustration human/ FN – Frustration non social) is FD (Frustration Dog) and the reference level for the factor sex is female.

**Table S4** – Results of the Model for the variable Proximity – stay close to the apparatus.

| **CONDITION** | **Df** | **AIC** | **BIC** | **logLik** | **deviance** | **Chisq** | **Df** | **Pr(>Chisq)** |
| --- | --- | --- | --- | --- | --- | --- | --- | --- |
| **null.CLOSE** | 10 | 3067.996 | 3113.363 | -1523.998 | 3047.996 | NA | NA | NA |
| **full.CLOSE** | 12 | 3000.929 | 3055.370 | -1488.465 | 2976.929 | 71.067 | 2 | 0.000 |

| **TRIAL** | **Df** | **AIC** | **BIC** | **logLik** | **deviance** | **Chisq** | **Df** | **Pr(>Chisq)** |
| --- | --- | --- | --- | --- | --- | --- | --- | --- |
| **null.CLOSE** | 8 | 3067.727 | 3104.020 | -1525.863 | 3051.727 | NA | NA | NA |
| **full.CLOSE** | 12 | 3000.929 | 3055.370 | -1488.465 | 2976.929 | 74.797 | 4 | 0.000 |

| **STAY CLOSE** | **Estimate** | **Std. Error** | **z-value** | **Pr(>\|z\|)** | **X2.5.** | **X97.5.** | **min** | **max** |
| --- | --- | --- | --- | --- | --- | --- | --- | --- |
| **(Intercept)** | 2.728 | 0.519 | 5.255 | 0.000 | 1.686 | 3.716 | 2.518 | 3.066 |
| **conditionFN** | 1.553 | 0.185 | 8.402 | **0.000** | 1.186 | 1.908 | 1.472 | 1.628 |
| **conditionFH** | 1.117 | 0.185 | 6.045 | **0.000** | 0.767 | 1.470 | 1.027 | 1.212 |
| **dog_typesheperd_type** | -0.368 | 0.346 | -1.066 | 0.287 | -1.055 | 0.315 | -0.488 | -0.251 |
| **sexm** | 0.218 | 0.340 | 0.641 | 0.521 | -0.407 | 0.822 | 0.118 | 0.354 |
| **trial2** | -1.106 | 0.239 | -4.634 | 0.000 | -1.576 | -0.668 | -1.164 | -1.031 |
| **trial3** | -1.341 | 0.239 | -5.618 | 0.000 | -1.787 | -0.882 | -1.409 | -1.277 |
| **trial4** | -1.893 | 0.239 | -7.932 | 0.000 | -2.330 | -1.398 | -1.966 | -1.836 |
| **trial5** | -1.758 | 0.239 | -7.367 | 0.000 | -2.177 | -1.284 | -1.836 | -1.681 |
| **age** | 0.028 | 0.082 | 0.343 | 0.731 | -0.121 | 0.179 | -0.012 | 0.073 |

# R2 for Mixed Models

Conditional R2: 0.337

Marginal R2: 0.157

**Simultaneous Tests for General Linear Hypotheses**

Multiple Comparisons of Means: Tukey Contrasts

Estimate Std. Error z-value Pr(>|z|)

FN - FD == 0 1.5530 0.1848 8.402 <1e-04 ***

FH - FD == 0 1.1174 0.1848 6.045 <1e-04 ***

FH - FN == 0 -0.4356 0.1848 -2.357 0.0483 *

**Table S5** – Results of the model for the variable Looking at the stimuli window.

| **CONDITION** | Df | AIC | BIC | logLik | deviance | Chisq | Df | Pr(>Chisq) |
| --- | --- | --- | --- | --- | --- | --- | --- | --- |
| **null.HSTIMULI** | 10 | 2867.643 | 2913.010 | -1423.822 | 2847.643 | NA | NA | NA |
| **full.HSTIMULI** | 12 | 2816.646 | 2871.086 | -1396.323 | 2792.646 | 54.998 | 2 | 0.000 |

| **TRIAL** | **Df** | **AIC** | **BIC** | **logLik** | **deviance** | **Chisq** | **Df** | **Pr(>Chisq)** |
| --- | --- | --- | --- | --- | --- | --- | --- | --- |
| **null.HSTIMULI** | 8 | 2847.184 | 2883.477 | -1415.592 | 2831.184 | NA | NA | NA |
| **full.HSTIMULI** | 12 | 2816.646 | 2871.086 | -1396.323 | 2792.646 | 38.538 | 4 | 0.000 |

|  | **Estimate** | **Std. Error** | **z-value** | **Pr(>\|z\|)** | **Lower CI** | **Upper CI** | **min** | **max** |
| --- | --- | --- | --- | --- | --- | --- | --- | --- |
| **(Intercept)** | 3.448 | 0.375 | 9.193 | 0.000 | 2.692 | 4.198 | 3.298 | 3.583 |
| **conditionFN** | 0.590 | 0.164 | 3.591 | **0.000** | 0.244 | 0.935 | 0.495 | 0.682 |
| **conditionFH** | 1.245 | 0.164 | 7.574 | **0.000** | 0.944 | 1.584 | 1.164 | 1.368 |
| **dog_typesheperd_type** | -0.299 | 0.240 | -1.247 | 0.213 | -0.783 | 0.185 | -0.367 | -0.230 |
| **sexm** | 0.332 | 0.237 | 1.403 | 0.161 | -0.154 | 0.770 | 0.252 | 0.410 |
| **trial2** | -0.841 | 0.212 | -3.960 | 0.000 | -1.266 | -0.410 | -0.916 | -0.791 |
| **trial3** | -0.794 | 0.212 | -3.742 | 0.000 | -1.176 | -0.404 | -0.830 | -0.736 |
| **trial4** | -1.003 | 0.212 | -4.725 | 0.000 | -1.424 | -0.596 | -1.055 | -0.920 |
| **trial5** | -1.262 | 0.212 | -5.947 | 0.000 | -1.692 | -0.848 | -1.319 | -1.181 |
| **age** | -0.050 | 0.057 | -0.874 | 0.382 | -0.161 | 0.057 | -0.070 | -0.015 |

Simultaneous Tests for General Linear Hypotheses

Multiple Comparisons of Means: Tukey Contrasts

Linear Hypotheses:

Estimate Std. Error z value Pr(>|z|)

FN - FD == 0 0.5904 0.1644 3.591 0.000924 ***

FH - FD == 0 1.2452 0.1644 7.574 < 1e-04 ***

FH - FN == 0 0.6548 0.1644 3.983 0.000200 ***

# R2 for Mixed Models

Conditional R2: 0.230

Marginal R2: 0.122

**Table S6** – Results for the model for the variable EAD101 – Ears Forward.

| **CONDITION** | **Df** | **AIC** | **BIC** | **logLik** | **deviance** | **Chisq** | **Df** | **Pr(>Chisq)** |
| --- | --- | --- | --- | --- | --- | --- | --- | --- |
| **null.EAD101B** | 9 | 829.556 | 870.386 | -405.778 | 811.556 | NA | NA | NA |
| **full.EAD101B** | 11 | 823.592 | 873.496 | -400.796 | 801.592 | 9.9640 | 2 | 0.007 |

| **TRIAL** | **Df** | **AIC** | **BIC** | **logLik** | **deviance** | **Chisq** | **Df** | **Pr(>Chisq)** |
| --- | --- | --- | --- | --- | --- | --- | --- | --- |
| **null.EAD101B** | 7 | 841.912 | 873.669 | -413.956 | 827.912 | NA | NA | NA |
| **full.EAD101B** | 11 | 823.592 | 873.496 | -400.796 | 801.592 | 26.319 | 4 | 0.000 |

|  | **Estimate** | **Std. Error** | **z-value** | **Pr(>\|z\|)** | **Lower CI** | **Upper CI** | **min** | **max** |
| --- | --- | --- | --- | --- | --- | --- | --- | --- |
| **(Intercept)** | 1.360 | 0.540 | 2.518 | 0.012 | 0.297 | 2.515 | 1.225 | 1.597 |
| **conditionFN** | 0.636 | 0.220 | 2.896 | **0.004** | 0.253 | 1.107 | 0.533 | 0.733 |
| **conditionFH** | 0.536 | 0.218 | 2.463 | **0.014** | 0.090 | 0.988 | 0.453 | 0.629 |
| **dog_typesheperd_type** | 0.331 | 0.347 | 0.956 | 0.339 | -0.383 | 1.036 | 0.234 | 0.453 |
| **sexm** | 0.199 | 0.342 | 0.582 | 0.560 | -0.442 | 0.877 | 0.087 | 0.313 |
| **trial2** | -0.762 | 0.304 | -2.504 | 0.012 | -1.373 | -0.214 | -0.883 | -0.689 |
| **trial3** | -0.841 | 0.303 | -2.772 | 0.006 | -1.454 | -0.269 | -0.966 | -0.731 |
| **trial4** | -1.218 | 0.301 | -4.050 | 0.000 | -1.853 | -0.641 | -1.319 | -1.122 |
| **trial5** | -1.363 | 0.301 | -4.534 | 0.000 | -1.927 | -0.814 | -1.470 | -1.273 |
| **age** | -0.085 | 0.082 | -1.044 | 0.297 | -0.246 | 0.075 | -0.154 | -0.041 |

Simultaneous Tests for General Linear Hypotheses

Multiple Comparisons of Means: Tukey Contrasts

Linear Hypotheses:

Estimate Std. Error z value Pr(>|z|)

FN - FD == 0 0.63583 0.21953 2.896 0.0107 *

FH - FD == 0 0.53606 0.21767 2.463 0.0368 *

FH - FN == 0 -0.09977 0.22341 -0.447 0.8959

# R2 for Mixed Models

Conditional R2: 0.286

Marginal R2: 0.081

**Table S7** – Results for the model for the variable EAD102 – Ears Adductor.

| **CONDITION** | **Df** | **AIC** | **BIC** | **logLik** | **deviance** | **Chisq** | **Df** | **Pr(>Chisq)** |
| --- | --- | --- | --- | --- | --- | --- | --- | --- |
| **null.EAD102B** | 9.000 | 849.156 | 889.986 | -415.578 | 831.156 | NA | NA | NA |
| **full.EAD102B** | 11 | 847.0248 | 896.9284 | -412.5124 | 825.0248 | 6.131231 | 2 | 0.047 |

| **TRIAL** | **Df** | **AIC** | **BIC** | **logLik** | **deviance** | **Chisq** | **Df** | **Pr(>Chisq)** |
| --- | --- | --- | --- | --- | --- | --- | --- | --- |
| **null.EAD102B** | 7.000 | 846.198 | 877.955 | -416.099 | 832.198 | NA | NA | NA |
| **full.EAD102B** | 11.000 | 847.025 | 896.928 | -412.512 | 825.025 | 7.173 | 4.000 | 0.127 |

|  | **Estimate** | **Std. Error** | **z value** | **Pr(>\|z\|)** | **Lower CI** | **Upper CI** | **min** | **max** |
| --- | --- | --- | --- | --- | --- | --- | --- | --- |
| **(Intercept)** | 0.581 | 0.494 | 1.176 | 0.240 | -0.333 | 1.645 | 0.381 | 0.744 |
| **conditionFN** | -0.399 | 0.218 | -1.834 | 0.067 | -0.833 | 0.001 | -0.509 | -0.313 |
| **conditionFH** | -0.510 | 0.217 | -2.350 | **0.019** | -0.964 | -0.113 | -0.646 | -0.406 |
| **dog_typesheperd_type** | 0.441 | 0.322 | 1.368 | 0.171 | -0.172 | 1.053 | 0.350 | 0.538 |
| **sexm** | 0.202 | 0.318 | 0.636 | 0.525 | -0.439 | 0.816 | 0.072 | 0.306 |
| **trial2** | 0.109 | 0.269 | 0.403 | 0.687 | -0.382 | 0.630 | 0.037 | 0.223 |
| **trial3** | 0.529 | 0.277 | 1.911 | 0.056 | -0.000 | 1.068 | 0.461 | 0.623 |
| **trial4** | 0.294 | 0.272 | 1.083 | 0.279 | -0.272 | 0.820 | 0.189 | 0.377 |
| **trial5** | 0.610 | 0.279 | 2.190 | 0.029 | 0.072 | 1.200 | 0.510 | 0.705 |
| **age** | -0.030 | 0.075 | -0.405 | 0.685 | -0.191 | 0.113 | -0.058 | -0.008 |

Simultaneous Tests for General Linear Hypotheses

Multiple Comparisons of Means: Tukey Contrasts

Linear Hypotheses:

Estimate Std. Error z value Pr(>|z|)

FN - FD == 0 -0.3992 0.2177 -1.834 0.1588

FH - FD == 0 -0.5099 0.2169 -2.350 0.0491 *

FH - FN == 0 -0.1106 0.2104 -0.526 0.8587

# R2 for Mixed Models

Conditional R2: 0.225

Marginal R2: 0.039

**Table S8** – Results for the model for the variable EAD103 – Ears Flattener.

| **CONDITION** | **Df** | **AIC** | **BIC** | **logLik** | **deviance** | **Chisq** | **Df** | **Pr(>Chisq)** |
| --- | --- | --- | --- | --- | --- | --- | --- | --- |
| **null.EAD103B** | 9 | 293.467 | 334.297 | -137.733 | 275.4669 | NA | NA | NA |
| **full.EAD103B** | 11 | 296.311 | 346.214 | -137.155 | 274.3108 | 1.156 | 2 | 0.561 |

| **TRIAL** | **Df** | **AIC** | **BIC** | **logLik** | **deviance** | **Chisq** | **Df** | **Pr(>Chisq)** |
| --- | --- | --- | --- | --- | --- | --- | --- | --- |
| **null.EAD103B** | 7 | 289.411 | 321.168 | -137.706 | 275.411 | NA | NA | NA |
| **full.EAD103B** | 11 | 296.311 | 346.214 | -137.155 | 274.311 | 1.101 | 4 | 0.894 |

|  | **Estimate** | **Std. Error** | **z value** | **Pr(>\|z\|)** | **Lower CI** | **Upper CI** | **min** | **max** |
| --- | --- | --- | --- | --- | --- | --- | --- | --- |
| **(Intercept)** | -4.135 | 1.219 | -3.391 | 0.001 | -8.201 | -1.892 | -5.137 | -3.809 |
| **conditionFN** | -0.459 | 0.433 | -1.060 | 0.289 | -1.578 | 0.422 | -0.926 | -0.203 |
| **conditionFH** | -0.259 | 0.417 | -0.622 | 0.534 | -1.267 | 0.629 | -0.829 | 0.000 |
| **dog_typesheperd_type** | 0.077 | 0.745 | 0.103 | 0.918 | -1.444 | 1.876 | -0.149 | 0.663 |
| **sexm** | 0.612 | 0.744 | 0.822 | 0.411 | -0.961 | 2.323 | 0.333 | 0.915 |
| **trial2** | -0.000 | 0.570 | -0.000 | 1.000 | -1.407 | 1.431 | -0.192 | 0.175 |
| **trial3** | 0.296 | 0.546 | 0.542 | 0.588 | -0.876 | 1.638 | 0.157 | 0.478 |
| **trial4** | -0.171 | 0.586 | -0.292 | 0.770 | -1.554 | 1.271 | -0.413 | 0.000 |
| **trial5** | 0.296 | 0.546 | 0.542 | 0.588 | -0.948 | 1.613 | 0.000 | 0.505 |
| **age** | -0.012 | 0.180 | -0.068 | 0.946 | -0.527 | 0.375 | -0.116 | 0.141 |

Simultaneous Tests for General Linear Hypotheses

Multiple Comparisons of Means: Tukey Contrasts

Linear Hypotheses:

Estimate Std. Error z-value Pr(>|z|)

FN - FD == 0 -0.4586 0.4326 -1.060 0.539

FH - FD == 0 -0.2594 0.4172 -0.622 0.808

FH - FN == 0 0.1992 0.4472 0.445 0.896

# R2 for Mixed Models

Conditional R2: 0.513

Marginal R2: 0.025

**Table S9** – Results for the model for the variable EAD104 – Ears Rotator

| **CONDITION** | **Df** | **AIC** | **BIC** | **logLik** | **deviance** | **Chisq** | **Df** | **Pr(>Chisq)** |
| --- | --- | --- | --- | --- | --- | --- | --- | --- |
| **null.EAD104B** | 9 | 884.245 | 925.075 | -433.122 | 866.245 | NA | NA | NA |
| **full.EAD104B** | 11 | 880.958 | 930.862 | -429.479 | 858.958 | 7.286 | 2 | 0.026 |

| **TRIAL** | **Df** | **AIC** | **BIC** | **logLik** | **deviance** | **Chisq** | **Df** | **Pr(>Chisq)** |
| --- | --- | --- | --- | --- | --- | --- | --- | --- |
| **null.EAD104B** | 7 | 878.329 | 910.086 | -432.165 | 864.328 | NA | NA | NA |
| **full.EAD104B** | 11 | 880.958 | 930.862 | -429.479 | 858.958 | 5.371 | 4 | 0.251 |

|  | **Estimate** | **Std. Error** | **z value** | **Pr(>\|z\|)** | **Lower CI** | **Upper CI** | **min** | **max** |
| --- | --- | --- | --- | --- | --- | --- | --- | --- |
| **(Intercept)** | -1.054 | 0.445 | -2.366 | 0.018 | -2.009 | -0.209 | -1.355 | -0.874 |
| **conditionFN** | -0.228 | 0.204 | -1.118 | 0.264 | -0.633 | 0.132 | -0.296 | -0.169 |
| **conditionFH** | -0.559 | 0.209 | -2.671 | 0.008 | -0.991 | -0.196 | -0.643 | -0.484 |
| **dog_typesheperd_type** | 0.482 | 0.279 | 1.729 | 0.084 | -0.032 | 1.062 | 0.364 | 0.604 |
| **sexm** | -0.012 | 0.273 | -0.044 | 0.965 | -0.581 | 0.529 | -0.140 | 0.076 |
| **trial2** | -0.075 | 0.275 | -0.275 | 0.784 | -0.583 | 0.502 | -0.155 | 0.039 |
| **trial3** | 0.460 | 0.267 | 1.720 | 0.085 | -0.040 | 1.022 | 0.397 | 0.510 |
| **trial4** | 0.253 | 0.269 | 0.939 | 0.348 | -0.258 | 0.809 | 0.149 | 0.333 |
| **trial5** | 0.288 | 0.269 | 1.071 | 0.284 | -0.219 | 0.824 | 0.221 | 0.372 |
| **age** | 0.048 | 0.066 | 0.731 | 0.465 | -0.085 | 0.190 | 0.006 | 0.089 |

Simultaneous Tests for General Linear Hypotheses

Multiple Comparisons of Means: Tukey Contrasts

Linear Hypotheses:

Estimate Std. Error z value Pr(>|z|)

FN - FD == 0 -0.2278 0.2038 -1.118 0.5030

FH - FD == 0 -0.5586 0.2091 -2.671 0.0208 *

FH - FN == 0 -0.3308 0.2107 -1.570 0.2587

# R2 for Mixed Models

Conditional R2: 0.169

Marginal R2: 0.039

**Table S10** – Results for the model for the variable EAD105 – Ears Downward.

| **CONDITION** | **Df** | **AIC** | **BIC** | **logLik** | **deviance** | **Chisq** | **Df** | **Pr(>Chisq)** |
| --- | --- | --- | --- | --- | --- | --- | --- | --- |
| **null.EAD105B** | 9 | 677.176 | 718.006 | -329.588 | 659.176 | NA | NA | NA |
| **full.EAD105B** | 11 | 674.433 | 724.337 | -326.217 | 652.433 | 6.743 | 2 | 0.034 |

| **TRIAL** | **Df** | **AIC** | **BIC** | **logLik** | **deviance** | **Chisq** | **Df** | **Pr(>Chisq)** |
| --- | --- | --- | --- | --- | --- | --- | --- | --- |
| **null.EAD105B** | 7 | 670.404 | 702.160 | -328.202 | 656.404 | NA | NA | NA |
| **full.EAD105B** | 11 | 674.433 | 724.337 | -326.217 | 652.433 | 3.970 | 4 | 0.409 |

|  | **Estimate** | **Std. Error** | **z value** | **Pr(>\|z\|)** | **Lower CI** | **Upper CI** | **min** | **max** |
| --- | --- | --- | --- | --- | --- | --- | --- | --- |
| **(Intercept)** | -1.198 | 0.570 | -2.102 | 0.036 | -2.369 | -0.132 | -1.400 | -0.937 |
| **conditionFN** | -0.628 | 0.254 | -2.474 | 0.013 | -1.169 | -0.118 | -0.720 | -0.515 |
| **conditionFH** | -0.146 | 0.242 | -0.603 | 0.546 | -0.634 | 0.352 | -0.273 | -0.030 |
| **dog_typesheperd_type** | -1.659 | 0.382 | -4.337 | 0.000 | -2.395 | -0.914 | -1.773 | -1.496 |
| **sexm** | -0.198 | 0.376 | -0.525 | 0.599 | -0.965 | 0.560 | -0.349 | -0.098 |
| **trial2** | 0.331 | 0.333 | 0.993 | 0.321 | -0.324 | 1.028 | 0.227 | 0.406 |
| **trial3** | 0.582 | 0.328 | 1.773 | 0.076 | -0.053 | 1.213 | 0.438 | 0.716 |
| **trial4** | 0.533 | 0.329 | 1.620 | 0.105 | -0.133 | 1.244 | 0.438 | 0.615 |
| **trial5** | 0.433 | 0.331 | 1.310 | 0.190 | -0.208 | 1.110 | 0.334 | 0.564 |
| **age** | 0.137 | 0.084 | 1.618 | 0.106 | -0.022 | 0.303 | 0.105 | 0.171 |

Simultaneous Tests for General Linear Hypotheses

Multiple Comparisons of Means: Tukey Contrasts

Linear Hypotheses:

Estimate Std. Error z value Pr(>|z|)

FN - FD == 0 -0.6277 0.2537 -2.474 0.0355 *

FH - FD == 0 -0.1457 0.2416 -0.603 0.8183

FH - FN == 0 0.4820 0.2554 1.887 0.1423

# R2 for Mixed Models

Conditional R2: 0.225

Marginal R2: 0.039

**Table S11** – Results for the model for the variable AD137 – Nose Lick.

| **CONDITION** | **Df** | **AIC** | **BIC** | **logLik** | **deviance** | **Chisq** | **Df** | **Pr(>Chisq)** |
| --- | --- | --- | --- | --- | --- | --- | --- | --- |
| **null.AD137B** | 9 | 527.861 | 568.691 | -254.930 | 509.861 | NA | NA | NA |
| **full.AD137B** | 11 | 525.729 | 575.633 | -251.865 | 503.729 | 6.132 | 2 | 0.047 |

| **TRIAL** | **Df** | **AIC** | **BIC** | **logLik** | **deviance** | **Chisq** | **Df** | **Pr(>Chisq)** |
| --- | --- | --- | --- | --- | --- | --- | --- | --- |
| **null.AD137B** | 7 | 520.271 | 552.028 | -253.136 | 506.271 | NA | NA | NA |
| **full.AD137B** | 11 | 525.729 | 575.633 | -251.865 | 503.729 | 2.542 | 4 | 0.637 |

|  | **Estimate** | **Std. Error** | **z value** | **Pr(>\|z\|)** | **Lower CI** | **Upper CI** | **min** | **max** |
| --- | --- | --- | --- | --- | --- | --- | --- | --- |
| **(Intercept)** | -1.864 | 0.532 | -3.503 | 0.000 | -3.110 | -0.906 | -2.009 | -1.691 |
| **conditionFN** | -0.645 | 0.290 | -2.226 | 0.026 | -1.267 | -0.103 | -0.756 | -0.506 |
| **conditionFH** | -0.548 | 0.284 | -1.929 | 0.054 | -1.111 | 0.000 | -0.618 | -0.451 |
| **dog_typesheperd_type** | -0.054 | 0.318 | -0.170 | 0.865 | -0.644 | 0.654 | -0.204 | 0.098 |
| **sexm** | 0.476 | 0.319 | 1.494 | 0.135 | -0.114 | 1.078 | 0.379 | 0.628 |
| **trial2** | -0.471 | 0.371 | -1.271 | 0.204 | -1.238 | 0.255 | -0.738 | -0.283 |
| **trial3** | -0.185 | 0.352 | -0.526 | 0.599 | -0.928 | 0.517 | -0.260 | -0.064 |
| **trial4** | -0.471 | 0.371 | -1.271 | 0.204 | -1.199 | 0.258 | -0.641 | -0.353 |
| **trial5** | -0.394 | 0.365 | -1.080 | 0.280 | -1.212 | 0.315 | -0.497 | -0.276 |
| **age** | 0.045 | 0.076 | 0.594 | 0.552 | -0.110 | 0.202 | -0.009 | 0.087 |

Simultaneous Tests for General Linear Hypotheses

Multiple Comparisons of Means: Tukey Contrasts

Linear Hypotheses:

Estimate Std. Error z-value Pr(>|z|)

FN - FD == 0 -0.64540 0.28997 -2.226 0.0668 .

FH - FD == 0 -0.54765 0.28386 -1.929 0.1301

FH - FN == 0 0.09775 0.31280 0.313 0.9475

# R2 for Mixed Models

Conditional R2: 0.157

Marginal R2: 0.047

**Table S12** – Results for the model for the variable AD37 – Lip Wipe.

| **CONDITION** | **Df** | **AIC** | **BIC** | **logLik** | **deviance** | **Chisq** | **Df** | **Pr(>Chisq)** |
| --- | --- | --- | --- | --- | --- | --- | --- | --- |
| **null.AD37B** | 9 | 617.669 | 664.738 | -299.835 | 599.669 | NA | NA | NA |
| **full.AD37B** | 11 | 608.712 | 666.240 | -293.356 | 586.712 | 12.957 | 2 | 0.002 |

| **TRIAL** | **Df** | **AIC** | **BIC** | **logLik** | **deviance** | **Chisq** | **Df** | **Pr(>Chisq)** |
| --- | --- | --- | --- | --- | --- | --- | --- | --- |
| **null.AD37B** | 7 | 603.270 | 639.879 | -294.635 | 589.270 | NA | NA | NA |
| **full.AD37B** | 11 | 608.712 | 666.240 | -293.356 | 586.712 | 2.558 | 4 | 0.634 |

|  | **Estimate** | **Std. Error** | **z value** | **Pr(>\|z\|)** | **Lower CI** | **Upper CI** | **min** | **max** |
| --- | --- | --- | --- | --- | --- | --- | --- | --- |
| **(Intercept)** | -2.522 | 0.515 | -4.897 | 0.000 | -3.708 | -1.601 | -2.717 | -2.322 |
| **conditionFN** | -0.876 | 0.289 | -3.025 | 0.002 | -1.515 | -0.326 | -1.001 | -0.793 |
| **conditionFH** | -0.821 | 0.285 | -2.882 | 0.004 | -1.441 | -0.274 | -0.998 | -0.708 |
| **dog_typesheperd_type** | -0.229 | 0.311 | -0.736 | 0.462 | -0.829 | 0.458 | -0.387 | -0.090 |
| **trial2** | -0.071 | 0.377 | -0.189 | 0.850 | -0.848 | 0.655 | -0.156 | 0.157 |
| **trial3** | -0.314 | 0.399 | -0.788 | 0.431 | -1.175 | 0.468 | -0.510 | -0.087 |
| **trial4** | 0.250 | 0.355 | 0.704 | 0.481 | -0.431 | 0.984 | 0.131 | 0.479 |
| **trial5** | 0.131 | 0.362 | 0.362 | 0.718 | -0.586 | 0.833 | 0.000 | 0.360 |
| **sexm** | 0.668 | 0.315 | 2.121 | 0.034 | 0.097 | 1.296 | 0.578 | 0.853 |
| **age** | -0.043 | 0.075 | -0.569 | 0.569 | -0.198 | 0.087 | -0.091 | 0.004 |

Simultaneous Tests for General Linear Hypotheses

Multiple Comparisons of Means: Tukey Contrasts

Linear Hypotheses:

Estimate Std. Error z-value Pr(>|z|)

FN - FD == 0 -0.87563 0.28944 -3.025 0.00689 **

FH - FD == 0 -0.82079 0.28484 -2.882 0.01129 *

FH - FN == 0 0.05483 0.33123 0.166 0.98492

# R2 for Mixed Models

Conditional R2: 0.180

Marginal R2: 0.081

**Table S13** – Results for the model for the variable AU145 – Blink.

| **trial** | **Df** | **AIC** | **BIC** | **logLik** | **deviance** | **Chisq** | **Df** | **Pr(>Chisq)** |
| --- | --- | --- | --- | --- | --- | --- | --- | --- |
| **null.AU145B** | 7 | 1837.448 | 1876.895 | -911.724 | 1823.448 | NA | NA | NA |
| **full.AU145B** | 11 | 1844.462 | 1906.451 | -911.231 | 1822.462 | 0.985 | 4 | 0.912 |

| **condition** | **Df** | **AIC** | **BIC** | **logLik** | **deviance** | **Chisq** | **Df** | **Pr(>Chisq)** |
| --- | --- | --- | --- | --- | --- | --- | --- | --- |
| **null.AU145B** | 9 | 1842.340 | 1893.058 | -912.170 | 1824.340 | NA | NA | NA |
| **full.AU145B** | 11 | 1844.462 | 1906.451 | -911.231 | 1822.462 | 1.878 | 2 | 0.391 |

|  | **Estimate** | **Std. Error** | **z value** | **Pr(>\|z\|)** | **Upper CI** | **Lower CI** | **min** | **max** |
| --- | --- | --- | --- | --- | --- | --- | --- | --- |
| **(Intercept)** | -1.880 | 0.241 | -7.798 | 0.000 | -2.402 | -1.424 | -1.977 | -1.753 |
| **conditionFN** | -0.052 | 0.144 | -0.360 | 0.719 | -0.345 | 0.241 | -0.097 | -0.011 |
| **conditionFH** | -0.196 | 0.148 | -1.326 | 0.185 | -0.508 | 0.091 | -0.247 | -0.146 |
| **dog_typesheperd_type** | 0.053 | 0.131 | 0.400 | 0.689 | -0.192 | 0.321 | 0.003 | 0.093 |
| **sexm** | 0.210 | 0.129 | 1.627 | 0.104 | -0.024 | 0.456 | 0.176 | 0.251 |
| **trial2** | 0.056 | 0.193 | 0.290 | 0.772 | -0.316 | 0.425 | 0.000 | 0.097 |
| **trial3** | 0.162 | 0.190 | 0.854 | 0.393 | -0.195 | 0.519 | 0.111 | 0.222 |
| **trial4** | 0.074 | 0.193 | 0.386 | 0.700 | -0.296 | 0.466 | 0.038 | 0.133 |
| **trial5** | 0.145 | 0.191 | 0.761 | 0.446 | -0.219 | 0.543 | 0.111 | 0.187 |
| **age** | 0.016 | 0.031 | 0.522 | 0.601 | -0.049 | 0.080 | 0.000 | 0.031 |

Simultaneous Tests for General Linear Hypotheses

Linear Hypotheses:

Estimate Std. Error z-value Pr(>|z|)

FN - FD == 0 -0.05195 0.14417 -0.360 0.931

FH - FD == 0 -0.19613 0.14791 -1.326 0.381

FH - FN == 0 -0.14418 0.14909 -0.967 0.598

# R2 for Mixed Models

Conditional R2: 0.015

Marginal R2: 0.007

**Table S14** – Results for the model for the variable AU101 – Inner Brow Raiser.

| **TRIAL** | **Df** | **AIC** | **BIC** | **logLik** | **deviance** | **Chisq** | **Df** | **Pr(>Chisq)** |
| --- | --- | --- | --- | --- | --- | --- | --- | --- |
| **null.AU101B** | 7.000 | 948.616 | 980.373 | -467.308 | 934.616 | NA | NA | NA |
| **full.AU101B** | 11.000 | 955.268 | 1005.172 | -466.634 | 933.268 | 1.348 | 4.000 | 0.853 |

| **CONDITION** | **Df** | **AIC** | **BIC** | **logLik** | **deviance** | **Chisq** | **Df** | **Pr(>Chisq)** |
| --- | --- | --- | --- | --- | --- | --- | --- | --- |
| **null.AU101B** | 9.000 | 955.055 | 995.885 | -468.527 | 937.055 | NA | NA | NA |
| **full.AU101B** | 11.000 | 955.268 | 1005.172 | -466.634 | 933.268 | 3.787 | 2.000 | 0.151 |

|  | **Estimate** | **Std. Error** | **z value** | **Pr(>\|z\|)** | **Lower CI** | **Upper CI** | **min** | **max** |
| --- | --- | --- | --- | --- | --- | --- | --- | --- |
| **(Intercept)** | -0.425 | 0.384 | -1.105 | 0.269 | -1.127 | 0.324 | -0.587 | -0.200 |
| **conditionFN** | 0.284 | 0.195 | 1.456 | 0.145 | -0.077 | 0.645 | 0.213 | 0.350 |
| **conditionFH** | 0.359 | 0.195 | 1.841 | 0.066 | -0.020 | 0.756 | 0.271 | 0.446 |
| **dog_typesheperd_type** | 0.065 | 0.235 | 0.279 | 0.780 | -0.394 | 0.512 | -0.019 | 0.161 |
| **sexm** | -0.092 | 0.231 | -0.398 | 0.691 | -0.561 | 0.347 | -0.167 | 0.005 |
| **trial2** | -0.220 | 0.251 | -0.878 | 0.380 | -0.725 | 0.280 | -0.291 | -0.161 |
| **trial3** | -0.220 | 0.251 | -0.878 | 0.380 | -0.719 | 0.259 | -0.291 | -0.161 |
| **trial4** | -0.252 | 0.251 | -1.003 | 0.316 | -0.775 | 0.221 | -0.323 | -0.192 |
| **trial5** | -0.126 | 0.251 | -0.502 | 0.616 | -0.619 | 0.388 | -0.194 | -0.064 |
| **age** | 0.069 | 0.057 | 1.218 | 0.223 | -0.035 | 0.188 | 0.010 | 0.104 |

Simultaneous Tests for General Linear Hypotheses

Multiple Comparisons of Means: Tukey Contrasts

Linear Hypotheses:

Estimate Std. Error z value Pr(>|z|)

FN - FD == 0 0.28392 0.19496 1.456 0.312

FH - FD == 0 0.35925 0.19509 1.841 0.156

FH - FN == 0 0.07532 0.19409 0.388 0.920

(Adjusted p values reported -- single-step method)

# R2 for Mixed Models

Conditional R2: 0.102

Marginal R2: 0.015

**Table S15** – Results for the model for the variable AD126 – Panting.

| **CONDITINO** | **Df** | **AIC** | **BIC** | **logLik** | **deviance** | **Chisq** | **Df** | **Pr(>Chisq)** |
| --- | --- | --- | --- | --- | --- | --- | --- | --- |
| **null.AD126B** | 9 | 304.735 | 345.565 | -143.367 | 286.735 | NA | NA | NA |
| **full.AD126B** | 11 | 274.508 | 324.412 | -126.254 | 252.508 | 34.226 | 2 | 0.000 |

| **TRIAL** | **Df** | **AIC** | **BIC** | **logLik** | **deviance** | **Chisq** | **Df** | **Pr(>Chisq)** |
| --- | --- | --- | --- | --- | --- | --- | --- | --- |
| **null.AD126B** | 7 | 267.875 | 299.632 | -126.937 | 253.875 | NA | NA | NA |
| **full.AD126B** | 11 | 274.508 | 324.412 | -126.254 | 252.508 | 1.367 | 4 | 0.850 |

|  | **Estimate** | **Std. Error** | **z value** | **Pr(>\|z\|)** | **X2.5.** | **X97.5.** | **min** | **max** |
| --- | --- | --- | --- | --- | --- | --- | --- | --- |
| **(Intercept)** | -7.080 | 2.566 | -2.760 | 0.006 | -17.159 | -2.571 | -8.547 | -6.330 |
| **conditionFN** | -2.580 | 0.536 | -4.811 | 0.000 | -4.946 | -1.637 | -3.211 | -2.243 |
| **conditionFH** | -1.978 | 0.496 | -3.991 | 0.000 | -3.842 | -1.026 | -2.463 | -1.593 |
| **dog_typesheperd_type** | 1.264 | 1.536 | 0.823 | 0.411 | -1.438 | 6.892 | 0.905 | 1.801 |
| **sexm** | 2.590 | 1.513 | 1.712 | 0.087 | -0.555 | 5.132 | 2.103 | 3.172 |
| **trial2** | 0.346 | 0.590 | 0.587 | 0.557 | -1.229 | 1.984 | 0.000 | 0.577 |
| **trial3** | -0.000 | 0.600 | -0.000 | 1.000 | -1.850 | 1.830 | -0.397 | 0.393 |
| **trial4** | 0.510 | 0.586 | 0.870 | 0.385 | -1.043 | 2.268 | 0.355 | 0.755 |
| **trial5** | -0.000 | 0.600 | -0.000 | 1.000 | -1.683 | 1.659 | -0.208 | 0.397 |
| **age** | -0.096 | 0.382 | -0.252 | 0.801 | -1.347 | 0.716 | -0.289 | 0.048 |

Simultaneous Tests for General Linear Hypotheses

Multiple Comparisons of Means: Tukey Contrasts

Linear Hypotheses:

Estimate Std. Error z value Pr(>|z|)

FN - FD == 0 -2.5805 0.5363 -4.811 0.000 ***

FH - FD == 0 -1.9780 0.4956 -3.991 0.000 ***

FH - FN == 0 0.6025 0.4960 1.215 0.444

# R2 for Mixed Models

Conditional R2: 0.876

Marginal R2: 0.129

**Table S16** - Model for the variable WHINE

| **TRIAL** | **Df** | **AIC** | **BIC** | **logLik** | **deviance** | **Chisq** | **Df** | **Pr(>Chisq)** |
| --- | --- | --- | --- | --- | --- | --- | --- | --- |
| **null.WHINEB** | 7 | 434.331 | 466.088 | -210.166 | 420.331 | NA | NA | NA |
| **full.WHINEB** | 11 | 439.509 | 489.412 | -208.754 | 417.509 | 2.823 | 4 | 0.588 |

| **CONDITION** | **Df** | **AIC** | **BIC** | **logLik** | **deviance** | **Chisq** | **Df** | **Pr(>Chisq)** |
| --- | --- | --- | --- | --- | --- | --- | --- | --- |
| **null.WHINEB** | 9 | 445.710 | 486.540 | -213.855 | 427.710 | NA | NA | NA |
| **full.WHINEB** | 11 | 439.509 | 489.412 | -208.754 | 417.509 | 10.201 | 2 | 0.006 |

|  | **Estimate** | **Std. Error** | **z value** | **Pr(>\|z\|)** | **Lower CI** | **Upper CI** | **min** | **max** |
| --- | --- | --- | --- | --- | --- | --- | --- | --- |
| **(Intercept)** | -1.916 | 0.963 | -1.990 | 0.047 | -4.076 | 0.055 | -2.171 | -1.410 |
| **conditionFN** | -0.822 | 0.329 | -2.502 | 0.012 | -1.566 | -0.199 | -1.049 | -0.702 |
| **conditionFH** | -0.950 | 0.336 | -2.829 | 0.005 | -1.711 | -0.318 | -1.151 | -0.832 |
| **dog_typesheperd_type** | -0.756 | 0.699 | -1.083 | 0.279 | -2.167 | 0.721 | -0.961 | -0.381 |
| **sexm** | 0.476 | 0.689 | 0.692 | 0.489 | -0.925 | 1.874 | 0.279 | 0.765 |
| **trial2** | 0.642 | 0.434 | 1.480 | 0.139 | -0.267 | 1.642 | 0.473 | 0.897 |
| **trial3** | 0.388 | 0.443 | 0.877 | 0.381 | -0.469 | 1.363 | 0.103 | 0.540 |
| **trial4** | 0.388 | 0.443 | 0.877 | 0.381 | -0.474 | 1.388 | 0.201 | 0.540 |
| **trial5** | 0.104 | 0.456 | 0.228 | 0.820 | -0.872 | 1.123 | -0.109 | 0.219 |
| **age** | -0.158 | 0.162 | -0.977 | 0.329 | -0.591 | 0.183 | -0.321 | -0.101 |

Simultaneous Tests for General Linear Hypotheses

Multiple Comparisons of Means: Tukey Contrasts

Linear Hypotheses:

Estimate Std. Error z value Pr(>|z|)

FN - FD == 0 -0.8221 0.3286 -2.502 0.0330 *

FH - FD == 0 -0.9496 0.3357 -2.829 0.0131 *

FH - FN == 0 -0.1275 0.3573 -0.357 0.9321

# R2 for Mixed Models

Conditional R2: 0.549

Marginal R2: 0.070

**Table S17** – Model for the variable BARK

| **CONDITION** | **Df** | **AIC** | **BIC** | **logLik** | **deviance** | **Chisq** | **Df** | **Pr(>Chisq)** |
| --- | --- | --- | --- | --- | --- | --- | --- | --- |
| **null.BARKB** | 9 | 273.080 | 313.910 | -127.540 | 255.080 | NA | NA | NA |
| **full.BARKB** | 11 | 273.932 | 323.835 | -125.966 | 251.932 | 3.148 | 2 | 0.207 |

| **TRIAL** | **Df** | **AIC** | **BIC** | **logLik** | **deviance** | **Chisq** | **Df** | **Pr(>Chisq)** |
| --- | --- | --- | --- | --- | --- | --- | --- | --- |
| **null.BARKB** | 7 | 270.098 | 301.854 | -128.049 | 256.0976 | NA | NA | NA |
| **full.BARKB** | 11 | 273.932 | 323.835 | -125.966 | 251.9318 | 4.166 | 4 | 0.384 |

|  | **Estimate** | **Std. Error** | **z value** | **Pr(>\|z\|)** | **Lower CI** | **Upper CI** | **min** | **max** |
| --- | --- | --- | --- | --- | --- | --- | --- | --- |
| **(Intercept)** | -4.245 | 1.802 | -2.355 | 0.019 | -12.377 | -0.749 | -5.329 | -3.544 |
| **conditionFN** | 0.434 | 0.469 | 0.926 | 0.355 | -0.687 | 1.817 | 0.121 | 1.048 |
| **conditionFH** | 0.796 | 0.457 | 1.744 | 0.081 | -0.197 | 2.230 | 0.344 | 1.430 |
| **dog_typesheperd_type** | -0.879 | 1.141 | -0.770 | 0.441 | -3.277 | 3.905 | -1.200 | -0.190 |
| **sexm** | -0.839 | 1.132 | -0.741 | 0.459 | -3.223 | 1.395 | -1.322 | -0.411 |
| **age** | -0.332 | 0.283 | -1.174 | 0.240 | -1.907 | 0.234 | -0.596 | -0.258 |
| **trial2** | 0.791 | 0.644 | 1.229 | 0.219 | -0.665 | 2.782 | 0.453 | 1.094 |
| **trial3** | 1.095 | 0.630 | 1.739 | 0.082 | -0.233 | 3.158 | 0.814 | 1.411 |
| **trial4** | 0.791 | 0.644 | 1.229 | 0.219 | -0.569 | 2.907 | 0.451 | 1.094 |
| **trial5** | 1.095 | 0.630 | 1.739 | 0.082 | -0.341 | 3.209 | 0.814 | 1.411 |

Simultaneous Tests for General Linear Hypotheses

Multiple Comparisons of Means: Tukey Contrasts

Linear Hypotheses:

Estimate Std. Error z value Pr(>|z|)

FN - FD == 0 0.4344 0.4694 0.926 0.624

FH - FD == 0 0.7961 0.4566 1.744 0.189

FH - FN == 0 0.3617 0.4273 0.846 0.674

# R2 for Mixed Models

Conditional R2: 0.759

Marginal R2: 0.083

**Table S18** – Model for the variable Head Turn.

| **TRIAL** | **Df** | **AIC** | **BIC** | **logLik** | **deviance** | **Chisq** | **Df** | **Pr(>Chisq)** |
| --- | --- | --- | --- | --- | --- | --- | --- | --- |
| **null.HEAD_TURNB** | 7 | 944.129 | 975.886 | -465.065 | 930.129 | NA | NA | NA |
| **full.HEAD_TURNB** | 11 | 949.334 | 999.237 | -463.667 | 927.334 | 2.795 | 4 | 0.593 |

| **CONDITION** | **Df** | **AIC** | **BIC** | **logLik** | **deviance** | **Chisq** | **Df** | **Pr(>Chisq)** |
| --- | --- | --- | --- | --- | --- | --- | --- | --- |
| **null.HEAD_TURNB** | 9 | 952.836 | 993.666 | -467.418 | 934.836 | NA | NA | NA |
| **full.HEAD_TURNB** | 11 | 949.334 | 999.237 | -463.667 | 927.334 | 7.502 | 2 | 0.023 |

|  | **Estimate** | **Std. Error** | **z value** | **Pr(>\|z\|)** | **Lower CI** | **Upper CI** | **min** | **max** |
| --- | --- | --- | --- | --- | --- | --- | --- | --- |
| **(Intercept)** | -0.537 | 0.345 | -1.557 | 0.119 | -1.225 | 0.102 | -0.778 | -0.365 |
| **conditionFN** | 0.110 | 0.191 | 0.574 | 0.566 | -0.272 | 0.455 | 0.019 | 0.187 |
| **conditionFH** | -0.396 | 0.195 | -2.031 | 0.042 | -0.804 | -0.038 | -0.464 | -0.327 |
| **dog_typesheperd_type** | 0.212 | 0.202 | 1.049 | 0.294 | -0.164 | 0.595 | 0.140 | 0.285 |
| **sexm** | -0.090 | 0.199 | -0.450 | 0.653 | -0.476 | 0.322 | -0.132 | -0.028 |
| **trial2** | 0.342 | 0.250 | 1.368 | 0.171 | -0.128 | 0.823 | 0.255 | 0.447 |
| **trial3** | 0.063 | 0.252 | 0.252 | 0.801 | -0.422 | 0.574 | 0.000 | 0.162 |
| **trial4** | 0.095 | 0.251 | 0.377 | 0.706 | -0.373 | 0.590 | 0.032 | 0.130 |
| **trial5** | 0.281 | 0.250 | 1.122 | 0.262 | -0.216 | 0.785 | 0.223 | 0.353 |
| **age** | 0.026 | 0.048 | 0.540 | 0.589 | -0.066 | 0.117 | -0.008 | 0.050 |

Simultaneous Tests for General Linear Hypotheses

Multiple Comparisons of Means: Tukey Contrasts

Fit: glmmTMB(formula = binomial ~ condition + dog_type + trial + age +

(1 | subject), data = HEAD_TURN_data, family = binomial(link = "logit"),

ziformula = ~0, dispformula = ~1)

Linear Hypotheses:

Estimate Std. Error z-value Pr(>|z|)

FN - FD == 0 0.1098 0.1914 0.574 0.8341

FH - FD == 0 -0.3962 0.1950 -2.031 0.1048

FH - FN == 0 -0.5060 0.1949 -2.597 0.0254 *

# R2 for Mixed Models

Conditional R2: 0.069

Marginal R2: 0.023

**Table S19** – Model for the variable sniffing the environment.

| **TRIAL** | **Df** | **AIC** | **BIC** | **logLik** | **deviance** | **Chisq** | **Df** | **Pr(>Chisq)** |
| --- | --- | --- | --- | --- | --- | --- | --- | --- |
| **null.SNIFFINGB** | 7 | 430.853 | 462.610 | -208.427 | 416.853 | NA | NA | NA |
| **full.SNIFFINGB** | 11 | 426.563 | 476.467 | -202.282 | 404.563 | 12.290 | 4 | 0.015 |

| **CONDITION** | **Df** | **AIC** | **BIC** | **logLik** | **deviance** | **Chisq** | **Df** | **Pr(>Chisq)** |
| --- | --- | --- | --- | --- | --- | --- | --- | --- |
| **null.SNIFFINGB** | 9 | 424.425 | 465.255 | -203.212 | 406.4245 | NA | NA | NA |
| **full.SNIFFINGB** | 11 | 426.563 | 476.467 | -202.282 | 404.563 | 1.861 | 2 | 0.394 |

|  | **Estimate** | **Std. Error** | **z value** | **Pr(>\|z\|)** | **Lower CI** | **Upper CI** | **min** | **max** |
| --- | --- | --- | --- | --- | --- | --- | --- | --- |
| **(Intercept)** | -2.591 | 0.671 | -3.863 | 0.000 | -4.232 | -1.446 | -2.903 | -2.380 |
| **conditionFN** | -0.407 | 0.344 | -1.183 | 0.237 | -1.180 | 0.284 | -0.656 | -0.249 |
| **conditionFH** | 0.000 | 0.318 | 0.000 | 1.000 | -0.639 | 0.696 | -0.218 | 0.110 |
| **dog_typesheperd_type** | -0.040 | 0.369 | -0.109 | 0.913 | -0.823 | 0.803 | -0.138 | 0.240 |
| **trial2** | 1.061 | 0.551 | 1.925 | 0.054 | -0.000 | 2.438 | 0.872 | 1.304 |
| **trial3** | 0.867 | 0.563 | 1.539 | 0.124 | -0.278 | 2.305 | 0.643 | 1.109 |
| **trial4** | 1.308 | 0.538 | 2.430 | 0.015 | 0.327 | 2.819 | 1.139 | 1.553 |
| **trial5** | 1.585 | 0.527 | 3.006 | 0.003 | 0.687 | 2.968 | 1.440 | 1.830 |
| **sexm** | -0.103 | 0.359 | -0.286 | 0.775 | -0.833 | 0.649 | -0.209 | 0.059 |
| **age** | -0.174 | 0.093 | -1.862 | 0.063 | -0.429 | 0.003 | -0.284 | -0.128 |

Simultaneous Tests for General Linear Hypotheses

Multiple Comparisons of Means: Tukey Contrasts

Linear Hypotheses:

Estimate Std. Error z-value Pr(>|z|)

FN - FD == 0 -4.071e-01 3.441e-01 -1.183 0.463

FH - FD == 0 1.488e-06 3.180e-01 0.000 1.000

FH - FN == 0 4.071e-01 3.441e-01 1.183 0.463

# R2 for Mixed Models

Conditional R2: 0.233

Marginal R2: 0.108

**Table S20** – Model for the variable paw lifting.

| **condition** | **Df** | **AIC** | **BIC** | **logLik** | **deviance** | **Chisq** | **Df** | **Pr(>Chisq)** |
| --- | --- | --- | --- | --- | --- | --- | --- | --- |
| **null.PAW_LIFTINGB** | 8 | 164.6836 | 200.9771 | -74.34179 | 148.6836 | NA | NA | NA |
| **full.PAW_LIFTINGB** | 10.000 | 163.672 | 209.039 | -71.836 | 143.6724 | 5.011207 | 2 | 0.08162634 |

| **condition** | **Df** | **AIC** | **BIC** | **logLik** | **deviance** | **Chisq** | **Df** | **Pr(>Chisq)** |
| --- | --- | --- | --- | --- | --- | --- | --- | --- |
| **null.PAW_LIFTINGB** | 6.000 | 165.058 | 192.278 | -76.529 | 153.058 | NA | NA | NA |
| **full.PAW_LIFTINGB** | 10.000 | 163.672 | 209.039 | -71.836 | 143.672 | 9.385 | 4.000 | 0.052 |

|  | **Estimate** | **Std. Error** | **z value** | **Pr(>\|z\|)** | **Lower CI** | **Upper CI** | **min** | **max** |
| --- | --- | --- | --- | --- | --- | --- | --- | --- |
| **(Intercept)** | -3.352 | 0.985 | -3.402 | 0.001 | -7.861 | -1.933 | -4.027 | -3.186 |
| **conditionFN** | -0.426 | 0.540 | -0.789 | 0.430 | -1.849 | 0.732 | -0.618 | -0.161 |
| **conditionFH** | -1.552 | 0.791 | -1.964 | 0.050 | -29.186 | -0.238 | -2.253 | -1.286 |
| **dog_typesheperd_type** | 0.399 | 0.555 | 0.719 | 0.472 | -0.619 | 2.696 | 0.225 | 0.749 |
| **sexm** | -0.400 | 0.504 | -0.792 | 0.428 | -1.666 | 0.729 | -0.530 | -0.207 |
| **trial2** | -0.725 | 0.722 | -1.004 | 0.315 | -22.256 | 0.755 | -1.143 | -0.531 |
| **trial3** | -30.991 | 2122016.000 | -0.000 | 1.000 | -44.580 | -19.601 | -34.578 | -26.153 |
| **trial4** | -0.193 | 0.623 | -0.310 | 0.757 | -1.676 | 1.417 | -0.429 | 0.000 |
| **trial5** | -0.725 | 0.722 | -1.004 | 0.315 | -24.601 | 0.737 | -0.142 | -0.531 |
| **age** | 0.143 | 0.128 | 1.116 | 0.265 | -0.150 | 0.597 | 0.117 | 0.235 |

Simultaneous Tests for General Linear Hypotheses

Multiple Comparisons of Means: Tukey Contrasts

Linear Hypotheses:

Estimate Std. Error z value Pr(>|z|)

FN - FD == 0 -0.4256 0.5397 -0.789 0.705

FH - FD == 0 -1.5507 0.7902 -1.962 0.118

FH - FN == 0 -1.1250 0.8246 -1.364 0.353

(Adjusted p values reported -- single-step method)

# R2 for Mixed Models

Conditional R2: NA

Marginal R2: 0.975

**Table S21** – Model for the variable Tail Wagging.

| **condition** | **Df** | **AIC** | **BIC** | **logLik** | **deviance** | **Chisq** | **Df** | **Pr(>Chisq)** |
| --- | --- | --- | --- | --- | --- | --- | --- | --- |
| **null.WAGB** | 9.000 | 525.881 | 566.712 | -253.941 | 507.8814 | NA | NA | NA |
| **full.WAGB** | 11 | 523.0613 | 572.9649 | -250.531 | 501.061 | 6.820 | 2.000 | 0.033 |

| **trial** | **Df** | **AIC** | **BIC** | **logLik** | **deviance** | **Chisq** | **Df** | **Pr(>Chisq)** |
| --- | --- | --- | --- | --- | --- | --- | --- | --- |
| **null.WAGB** | 7 | 519.6532 | 551.4100 | -252.827 | 505.6532 | NA | NA | NA |
| **full.WAGB** | 11 | 523.0613 | 572.9649 | -250.5306 | 501.0613 | 4.591941 | 4 | 0.332 |

|  | **Estimate** | **Std. Error** | **z value** | **Pr(>\|z\|)** | **Lower CI** | **Upper CI** | **min** | **max** |
| --- | --- | --- | --- | --- | --- | --- | --- | --- |
| **(Intercept)** | -0.592 | 0.839 | -0.706 | 0.480 | -2.309 | 1.024 | -0.934 | -0.100 |
| **conditionFN** | -0.730 | 0.307 | -2.374 | 0.018 | -1.389 | -0.149 | -1.035 | -0.531 |
| **conditionFH** | -0.082 | 0.287 | -0.286 | 0.775 | -0.709 | 0.473 | -0.260 | 0.087 |
| **dog_typesheperd_type** | -2.087 | 0.599 | -3.485 | 0.000 | -3.325 | -0.875 | -2.285 | -1.802 |
| **sexm** | 0.082 | 0.589 | 0.140 | 0.889 | -1.145 | 1.283 | -0.129 | 0.342 |
| **trial2** | -0.573 | 0.382 | -1.501 | 0.133 | -1.373 | 0.184 | -0.672 | -0.506 |
| **trial3** | -0.203 | 0.368 | -0.551 | 0.582 | -0.908 | 0.543 | -0.288 | -0.138 |
| **trial4** | -0.345 | 0.373 | -0.927 | 0.354 | -1.113 | 0.405 | -0.443 | -0.217 |
| **trial5** | -0.736 | 0.389 | -1.891 | 0.059 | -1.581 | 0.002 | -0.870 | -0.617 |
| **age** | 0.004 | 0.131 | 0.030 | 0.976 | -0.275 | 0.256 | -0.079 | 0.071 |

Simultaneous Tests for General Linear Hypotheses

Multiple Comparisons of Means: Tukey Contrasts

Linear Hypotheses:

Estimate Std. Error z value Pr(>|z|)

FN - FD == 0 -0.72989 0.30745 -2.374 0.0462 *

FH - FD == 0 -0.08208 0.28657 -0.286 0.9557

FH - FN == 0 0.64781 0.30850 2.100 0.0897 .

# R2 for Mixed Models

Conditional R2: 0.547

Marginal R2: 0.170

**Table S22 – Results for the model for the Post-cortisol concentrations.**
In all the models the the reference level for the Fixed factor **Condition** (FD- Frustration dog/FH – Frustration human/ FN – Frustration non social) is FD (Frustration Dog) and the reference level for the factor sex is female.

| **condition** | **Df** | **AIC** | **BIC** | **logLik** | **deviance** | **Chisq** | **Df** | **Pr(>Chisq)** |
| --- | --- | --- | --- | --- | --- | --- | --- | --- |
| **null.cortisol** | 7.000 | 467.883 | 487.337 | -226.941 | 453.883 | NA | NA | NA |
| **full.cortisol** | 9.000 | 471.662 | 496.674 | -226.831 | 453.662 | 0.221 | 2.000 | 0.895 |

|  | Df | AIC | LRT | Pr(>Chi) |
| --- | --- | --- | --- | --- |
| **<none>** | NA | 471.662 | NA | NA |
| **condition** | 2 | 467.883 | 0.221 | 0.895 |
| **pre_c** | 1 | 575.822 | 106.160 | 0.000 |
| **sex** | 1 | 469.698 | 0.036 | 0.849 |
| **age** | 1 | 470.418 | 0.756 | 0.384 |

| **Post-test cortisol** | **Estimate** | **Std. Error** | **z value** | **P-value** | **Lower CI** | **Upper CI** | **min** | **max** |
| --- | --- | --- | --- | --- | --- | --- | --- | --- |
| **(Intercept)** | -0.445 | 0.515 | -0.864 | 0.388 | -1.434 | 0.573 | -0.603 | -0.285 |
| **conditionFN** | 0.143 | 0.340 | 0.420 | 0.674 | -0.476 | 0.800 | 0.143 | 0.245 |
| **conditionFH** | 0.009 | 0.338 | 0.026 | 0.979 | -0.610 | 0.639 | -0.030 | 0.091 |
| **pre_c** | 0.941 | 0.067 | 14.101 | **0.000** | 0.822 | 1.087 | 0.941 | 1.090 |
| **sexm** | -0.068 | 0.356 | -0.191 | 0.848 | -0.842 | 0.622 | -0.090 | -0.032 |
| **age** | 0.074 | 0.085 | 0.874 | 0.382 | -0.083 | 0.246 | -0.023 | 0.088 |

**Table S23** – Association between Pre-test Cortisol concentration and EAD 101 – Ears Forward.

| **EAD101 – Ears Forward** | **Estimate** | **Std. Error** | **z value** | **Pr(>\|z\|)** | **Lower CI** | **Upper CI** | **min** | **max** |
| --- | --- | --- | --- | --- | --- | --- | --- | --- |
| **(Intercept)** | 1.695 | 0.490 | 3.455 | 0.001 | 0.668 | 2.684 | 1.334 | 1.905 |
| **pre_c** | 0.029 | 0.043 | 0.676 | 0.499 | -0.056 | 0.108 | 0.008 | 0.047 |
| **conditionFN** | 0.523 | 0.173 | 3.023 | 0.003 | 0.215 | 0.865 | 0.422 | 0.722 |
| **conditionFH** | 0.618 | 0.173 | 3.568 | 0.000 | 0.311 | 0.960 | 0.517 | 0.690 |
| **sexm** | 0.097 | 0.340 | 0.285 | 0.776 | -0.591 | 0.785 | -0.069 | 0.268 |
| **age** | -0.035 | 0.080 | -0.440 | 0.660 | -0.202 | 0.121 | -0.095 | 0.022 |
| **dog_typesheperd_type** | -0.218 | 0.335 | -0.649 | 0.516 | -0.891 | 0.434 | -0.343 | -0.042 |

**Table S24** – Association between Pre-test Cortisol concentration and EAD102 – Ears Adductor.

| **EAD102 - Ears Adductor** | **Estimate** | **Std. Error** | **z value** | **Pr(>\|z\|)** | **Lower CI** | **Upper CI** | **min** | **max** |
| --- | --- | --- | --- | --- | --- | --- | --- | --- |
| **(Intercept)** | 1.613 | 0.374 | 4.314 | 0.000 | 0.908 | 2.313 | 1.281 | 1.974 |
| **pre_c** | 0.033 | 0.036 | 0.921 | 0.357 | -0.036 | 0.104 | 0.001 | 0.139 |
| **conditionFN** | -0.230 | 0.155 | -1.483 | 0.138 | -0.539 | 0.070 | -0.350 | -0.137 |
| **conditionFH** | -0.026 | 0.155 | -0.165 | 0.869 | -0.315 | 0.286 | -0.156 | 0.234 |
| **sexm** | 0.131 | 0.257 | 0.511 | 0.609 | -0.415 | 0.681 | -0.066 | 0.290 |
| **age** | -0.072 | 0.060 | -1.198 | 0.231 | -0.188 | 0.049 | -0.090 | -0.036 |
| **dog_typesheperd_type** | 0.273 | 0.252 | 1.084 | 0.279 | -0.250 | 0.785 | 0.126 | 0.439 |

**Table S25** – Association between Pre-test Cortisol concentration and EAD103 – Ears Flattener.

| **EAD103 - Ears flattener** | **Estimate** | **Std. Error** | **z-value** | **Pr(>\|z\|)** | **Lower CI** | **Upper CI** | **min** | **max** |
| --- | --- | --- | --- | --- | --- | --- | --- | --- |
| **(Intercept)** | 0.082 | 0.072 | 1.136 | 0.256 | -0.056 | 0.215 | 0.039 | 0.125 |
| **pre_c** | -0.001 | 0.007 | -0.202 | 0.840 | -0.014 | 0.011 | -0.004 | 0.002 |
| **conditionFN** | -0.018 | 0.030 | -0.584 | 0.559 | -0.076 | 0.041 | -0.074 | 0.010 |
| **conditionFH** | -0.008 | 0.030 | -0.251 | 0.802 | -0.063 | 0.049 | -0.029 | 0.018 |
| **sexm** | 0.033 | 0.049 | 0.678 | 0.498 | -0.051 | 0.116 | 0.023 | 0.048 |
| **age** | -0.005 | 0.011 | -0.512 | 0.609 | -0.026 | 0.015 | -0.012 | 0.003 |
| **dog_typesheperd_type** | 0.012 | 0.042 | 0.288 | 0.773 | -0.073 | 0.097 | -0.008 | 0.024 |

**Table S26** – Association between Pre-test Cortisol concentration and EAD104 – Ears Rotator.

| **EAD104 - Ears rotator** | **Estimate** | **Std. Error** | **z-value** | **Pr(>\|z\|)** | **Lower CI** | **Upper CI** | **min** | **max** |
| --- | --- | --- | --- | --- | --- | --- | --- | --- |
| **(Intercept)** | 0.221 | 0.227 | 0.974 | 0.330 | -0.243 | 0.669 | 0.137 | 0.290 |
| **pre_c** | -0.009 | 0.021 | -0.411 | 0.681 | -0.046 | 0.032 | -0.030 | 0.004 |
| **conditionFN** | 0.001 | 0.083 | 0.015 | 0.988 | -0.164 | 0.168 | -0.063 | 0.048 |
| **conditionFH** | -0.145 | 0.083 | -1.750 | 0.080 | -0.315 | 0.025 | -0.188 | -0.090 |
| **sexm** | -0.018 | 0.157 | -0.117 | 0.907 | -0.320 | 0.262 | -0.100 | 0.046 |
| **age** | 0.033 | 0.037 | 0.895 | 0.371 | -0.036 | 0.105 | -0.005 | 0.043 |
| **dog_typesheperd_type** | 0.354 | 0.155 | 2.288 | 0.022 | 0.048 | 0.650 | 0.259 | 0.427 |

**Table S27** – Association between Pre-test Cortisol concentration and EAD105 – Ears Downward.

| **EAD105 - ears downward** | **Estimate** | **Std. Error** | **z-value** | **Pr(>\|z\|)** | **Lower CI** | **Upper CI** | **min** | **max** |
| --- | --- | --- | --- | --- | --- | --- | --- | --- |
| **(Intercept)** | 1.126 | 0.214 | 5.254 | 0.000 | 0.717 | 1.566 | 1.006 | 1.193 |
| **pre_c** | -0.040 | 0.023 | -1.762 | 0.078 | -0.085 | 0.005 | -0.069 | -0.019 |
| **conditionFN** | -0.337 | 0.105 | -3.215 | 0.001 | -0.537 | -0.149 | -0.464 | -0.288 |
| **conditionFH** | -0.283 | 0.105 | -2.694 | 0.007 | -0.495 | -0.077 | -0.317 | -0.238 |
| **sexm** | -0.231 | 0.145 | -1.598 | 0.110 | -0.488 | 0.066 | -0.288 | -0.110 |
| **age** | 0.051 | 0.034 | 1.515 | 0.130 | -0.022 | 0.119 | 0.019 | 0.086 |
| **dog_typesheperd_type** | -0.699 | 0.142 | -4.917 | 0.000 | -0.987 | -0.419 | -0.802 | -0.647 |

**Table S28** - Association between Pre-test Cortisol concentration and AD137 – Nose lick.

| **AD137 – Nose lick** | **Estimate** | **Std. Error** | **z-value** | **Pr(>\|z\|)** | **Lower CI** | **Upper CI** | **min** | **max** |
| --- | --- | --- | --- | --- | --- | --- | --- | --- |
| **(Intercept)** | -2.125 | 0.493 | -4.307 | 0.000 | -3.325 | -1.176 | -2.332 | -1.988 |
| **pre_c** | -0.026 | 0.050 | -0.516 | 0.606 | -0.196 | 0.053 | -0.043 | -0.016 |
| **conditionFN** | -0.532 | 0.257 | -2.068 | 0.039 | -1.095 | -0.030 | -0.764 | -0.375 |
| **conditionFH** | -0.542 | 0.257 | -2.107 | 0.035 | -1.076 | -0.028 | -0.714 | -0.367 |
| **sexm** | 0.221 | 0.311 | 0.710 | 0.478 | -0.367 | 0.864 | 0.029 | 0.379 |
| **age** | 0.068 | 0.071 | 0.961 | 0.337 | -0.079 | 0.225 | 0.025 | 0.117 |
| **dog_typesheperd_type** | 0.019 | 0.340 | 0.055 | 0.956 | -0.586 | 0.697 | -0.122 | 0.161 |

**Table S29** – Association between Pre-test Cortisol concentration and AD137 – Lip Wipe.

| **AD37 - Lip wipe** | **Estimate** | **Std. Error** | **z value** | **Pr(>\|z\|)** | **Lower CI** | **Upper CI** | **min** | **max** |
| --- | --- | --- | --- | --- | --- | --- | --- | --- |
| **(Intercept)** | 0.019 | 0.385 | 0.050 | 0.960 | -0.860 | 0.770 | -0.484 | 0.379 |
| **pre_c** | -0.036 | 0.050 | -0.724 | 0.469 | -0.191 | 0.033 | -0.083 | -0.011 |
| **conditionFN** | -0.934 | 0.295 | -3.166 | 0.002 | -1.560 | -0.387 | -1.191 | -0.832 |
| **conditionFH** | -0.829 | 0.283 | -2.933 | 0.003 | -1.480 | -0.324 | -1.110 | -0.725 |
| **sexm** | 0.654 | 0.282 | 2.323 | 0.020 | 0.091 | 1.270 | 0.520 | 0.838 |
| **age** | -0.030 | 0.059 | -0.516 | 0.606 | -0.159 | 0.081 | -0.089 | 0.038 |
| **dog_typesheperd_type** | -0.438 | 0.257 | -1.707 | 0.088 | -0.953 | 0.100 | -0.768 | -0.164 |

**Table S30** – Association between Pre-test Cortisol concentration and for AU145 – Blink.

| **AU145 - Blink** | **Estimate** | **Std. Error** | **z value** | **Pr(>\|z\|)** | **Lower CI** | **Upper CI** | **min** | **max** |
| --- | --- | --- | --- | --- | --- | --- | --- | --- |
| **(Intercept)** | -0.570 | 0.266 | -2.140 | 0.032 | -1.094 | -0.037 | -0.681 | -0.306 |
| **pre_c** | -0.005 | 0.024 | -0.211 | 0.833 | -0.063 | 0.040 | -0.018 | 0.008 |
| **conditionFN** | -0.006 | 0.118 | -0.052 | 0.958 | -0.216 | 0.223 | -0.055 | 0.071 |
| **conditionFH** | -0.152 | 0.123 | -1.234 | 0.217 | -0.383 | 0.093 | -0.346 | -0.021 |
| **sexm** | 0.076 | 0.183 | 0.418 | 0.676 | -0.227 | 0.399 | -0.059 | 0.171 |
| **age** | 0.030 | 0.041 | 0.750 | 0.453 | -0.051 | 0.107 | -0.010 | 0.048 |
| **dog_typesheperd_type** | 0.014 | 0.176 | 0.081 | 0.935 | -0.306 | 0.346 | -0.109 | 0.100 |

**Table S31** – Association between Pre-test Cortisol concentration and AU101 - Inner Brow Raiser.

| **AU101 – Inner Brow Raiser** | **Estimate** | **Std. Error** | **Z-value** | **Pr(>\|z\|)** | **Lower CI** | **Upper CI** | **min** | **max** |
| --- | --- | --- | --- | --- | --- | --- | --- | --- |
| **(Intercept)** | 0.442 | 0.194 | 2.286 | 0.022 | 0.055 | 0.804 | 0.106 | 0.546 |
| **pre_c** | -0.017 | 0.019 | -0.881 | 0.378 | -0.051 | 0.023 | -0.047 | 0.004 |
| **conditionFN** | 0.123 | 0.093 | 1.321 | 0.187 | -0.060 | 0.289 | 0.045 | 0.175 |
| **conditionFH** | 0.302 | 0.093 | 3.235 | 0.001 | 0.130 | 0.484 | 0.198 | 0.367 |
| **sexm** | -0.175 | 0.123 | -1.422 | 0.155 | -0.402 | 0.048 | -0.319 | -0.055 |
| **age** | 0.046 | 0.028 | 1.611 | 0.107 | -0.008 | 0.104 | 0.030 | 0.105 |
| **dog_typesheperd_type** | 0.129 | 0.115 | 1.126 | 0.260 | -0.087 | 0.354 | 0.052 | 0.218 |

**Table S32** – Association between Pre-test Cortisol concentration and Panting.

| **AD126 - Panting** | **Estimate** | **Std. Error** | **value** | **Pr(>\|z\|)** | **Lower CI** | **Upper CI** | **min** | **max** |
| --- | --- | --- | --- | --- | --- | --- | --- | --- |
| **(Intercept)** | 0.219 | 0.317 | 0.690 | 0.490 | -0.393 | 0.859 | 0.171 | 0.288 |
| **pre_c** | 0.032 | 0.025 | 1.278 | 0.201 | -0.018 | 0.079 | 0.015 | 0.041 |
| **conditionFN** | -0.378 | 0.097 | -3.905 | 0.000 | -0.562 | -0.183 | -0.539 | -0.268 |
| **conditionFH** | -0.366 | 0.097 | -3.763 | 0.000 | -0.554 | -0.176 | -0.434 | -0.306 |
| **sexm** | 0.146 | 0.221 | 0.658 | 0.511 | -0.296 | 0.567 | 0.040 | 0.338 |
| **age** | 0.003 | 0.052 | 0.049 | 0.961 | -0.097 | 0.102 | -0.010 | 0.008 |
| **dog_typesheperd_type** | 0.446 | 0.218 | 2.043 | 0.041 | -0.007 | 0.868 | 0.333 | 0.553 |

**Table S33** – Association between Pre-test Cortisol concentration and Tail wagging.

| **TAIL WAGGING** | **Estimate** | **Std. Error** | **z-value** | **Pr(>\|z\|)** | **Lower CI** | **Upper CI** | **min** | **max** |
| --- | --- | --- | --- | --- | --- | --- | --- | --- |
| **(Intercept)** | 1.579 | 0.513 | 3.077 | 0.002 | 0.643 | 2.508 | 0.662 | 2.066 |
| **pre_c** | -0.047 | 0.034 | -1.397 | 0.162 | -0.112 | 0.024 | -0.081 | -0.017 |
| **conditionFN** | -0.216 | 0.129 | -1.677 | 0.094 | -0.470 | 0.047 | -0.453 | -0.116 |
| **conditionFH** | 0.047 | 0.128 | 0.369 | 0.712 | -0.211 | 0.311 | -0.198 | 0.166 |
| **sexm** | -0.188 | 0.338 | -0.557 | 0.578 | -0.863 | 0.442 | -0.374 | 0.156 |
| **age** | 0.021 | 0.082 | 0.255 | 0.799 | -0.130 | 0.177 | -0.140 | 0.130 |
| **dog_typesheperd_type** | -1.109 | 0.335 | -3.307 | 0.001 | -1.789 | -0.467 | -1.271 | -0.805 |

**Table S34** – Association between Pre-test Cortisol concentration and Sniffing the environment.

| **SNIFFING THE ENVIRONMENT** | **Estimate** | **Std. Error** | **value** | **Pr(>\|z\|)** | **Lower CI** | **Upper CI** | **min** | **max** |
| --- | --- | --- | --- | --- | --- | --- | --- | --- |
| **(Intercept)** | 0.327 | 0.129 | 2.544 | 0.011 | 0.083 | 0.582 | 0.254 | 0.385 |
| **pre_c** | -0.011 | 0.015 | -0.741 | 0.458 | -0.040 | 0.018 | -0.019 | -0.006 |
| **conditionFN** | -0.148 | 0.082 | -1.804 | 0.071 | -0.307 | 0.020 | -0.204 | -0.049 |
| **conditionFH** | 0.003 | 0.082 | 0.038 | 0.970 | -0.165 | 0.163 | -0.055 | 0.089 |
| **sexm** | 0.033 | 0.082 | 0.401 | 0.689 | -0.133 | 0.210 | -0.014 | 0.090 |
| **age** | -0.011 | 0.019 | -0.555 | 0.579 | -0.049 | 0.030 | -0.033 | 0.002 |
| **dog_typesheperd_type** | -0.025 | 0.080 | -0.312 | 0.755 | -0.182 | 0.126 | -0.078 | 0.035 |

**Table S35** – Association between Pre-test Cortisol concentration and for Head turn.

| **HEAD TURN** | **Estimate** | **Std. Error** | **z-value** | **Pr(>\|z\|)** | **Lower CI** | **Upper CI** | **min** | **max** |
| --- | --- | --- | --- | --- | --- | --- | --- | --- |
| **(Intercept)** | -0.738 | 0.224 | -3.292 | 0.001 | -1.163 | -0.320 | -0.919 | -0.570 |
| **pre_c** | -0.013 | 0.025 | -0.500 | 0.617 | -0.080 | 0.031 | -0.088 | 0.003 |
| **conditionFN** | -0.055 | 0.133 | -0.412 | 0.680 | -0.333 | 0.219 | -0.075 | -0.022 |
| **conditionFH** | -0.414 | 0.147 | -2.815 | 0.005 | -0.708 | -0.120 | -0.451 | -0.327 |
| **sexm** | -0.011 | 0.144 | -0.076 | 0.939 | -0.292 | 0.275 | -0.090 | 0.066 |
| **age** | 0.030 | 0.034 | 0.866 | 0.387 | -0.039 | 0.098 | 0.006 | 0.060 |
| **dog_typesheperd_type** | 0.157 | 0.143 | 1.102 | 0.271 | -0.115 | 0.444 | 0.032 | 0.259 |

**Table S36** – Association between Pre-test Cortisol concentration and for Whine.

| **WHINE** | **Estimate** | **Std. Error** | **z-value** | **Pr(>\|z\|)** | **Lower CI** | **Upper CI** | **min** | **max** |
| --- | --- | --- | --- | --- | --- | --- | --- | --- |
| **(Intercept)** | 0.262 | 0.116 | 2.265 | 0.023 | 0.028 | 0.503 | 0.130 | 0.325 |
| **pre_c** | -0.003 | 0.011 | -0.233 | 0.816 | -0.025 | 0.019 | -0.007 | 0.011 |
| **conditionFN** | -0.118 | 0.050 | -2.381 | 0.017 | -0.218 | -0.021 | -0.169 | -0.069 |
| **conditionFH** | -0.137 | 0.050 | -2.757 | 0.006 | -0.238 | -0.034 | -0.159 | -0.098 |
| **sexm** | 0.040 | 0.079 | 0.502 | 0.615 | -0.116 | 0.199 | 0.001 | 0.078 |
| **age** | -0.003 | 0.019 | -0.171 | 0.864 | -0.039 | 0.032 | -0.022 | 0.015 |
| **dog_typesheperd_type** | -0.017 | 0.078 | -0.217 | 0.828 | -0.163 | 0.136 | -0.039 | 0.030 |

**Table S37** – Association between Pre-test Cortisol concentration and Proximity to the apparatus.

| **CLOSE - Proximity to apparatus** | **Estimate** | **Std. Error** | **z value** | **Pr(>\|z\|)** | **Lower CI** | **Upper CI** | **min** | **max** |
| --- | --- | --- | --- | --- | --- | --- | --- | --- |
| **(Intercept)** | 1.782 | 0.544 | 3.277 | 0.001 | 0.661 | 2.857 | 1.300 | 2.493 |
| **pre_c** | 0.042 | 0.051 | 0.815 | 0.415 | -0.065 | 0.139 | 0.015 | 0.073 |
| **conditionFN** | 1.500 | 0.215 | 6.976 | 0.000 | 1.074 | 1.919 | 1.337 | 1.666 |
| **conditionFH** | 1.066 | 0.215 | 4.947 | 0.000 | 0.653 | 1.532 | 0.883 | 1.284 |
| **sexm** | -0.072 | 0.375 | -0.192 | 0.848 | -0.802 | 0.587 | -0.280 | 0.115 |
| **age** | 0.034 | 0.088 | 0.384 | 0.701 | -0.130 | 0.221 | -0.037 | 0.071 |
| **dog_typesheperd_type** | -0.509 | 0.368 | -1.381 | 0.167 | -1.264 | 0.195 | -0.781 | -0.316 |

**Table S38** – Association between Pre-test Cortisol concentration and Looking at the apparatus.

| **LOOKING APPARATUS** | **Estimate** | **Std. Error** | **z-value** | **Pr(>\|z\|)** | **Lower CI** | **Upper CI** | **min** | **max** |
| --- | --- | --- | --- | --- | --- | --- | --- | --- |
| **(Intercept)** | 2.577 | 0.378 | 6.821 | 0.000 | 1.843 | 3.332 | 2.359 | 2.782 |
| **pre_c** | -0.013 | 0.040 | -0.320 | 0.749 | -0.089 | 0.070 | -0.028 | 0.066 |
| **conditionFN** | 0.675 | 0.185 | 3.647 | 0.000 | 0.312 | 1.026 | 0.528 | 0.751 |
| **conditionFH** | 1.260 | 0.185 | 6.800 | 0.000 | 0.902 | 1.609 | 1.147 | 1.421 |
| **sexm** | 0.359 | 0.255 | 1.406 | 0.160 | -0.135 | 0.876 | 0.119 | 0.674 |
| **age** | -0.023 | 0.060 | -0.376 | 0.707 | -0.145 | 0.100 | -0.041 | 0.033 |
| **dog_typesheperd_type** | -0.399 | 0.250 | -1.592 | 0.111 | -0.923 | 0.076 | -0.570 | -0.229 |
